# Immunometabolic reprogramming in osteoporosis–osteoarthritis comorbidity: from inflammaging to osteochondral unit degeneration

**DOI:** 10.3389/fimmu.2026.1941310

**Published:** 2026-09-18

**Authors:** Wenxiong Li, Jie Liu, Jiao Li, Feng Yang, Weiwei Huang

**Affiliations:** 1 First Clinical Medical College, Shaanxi University of Chinese Medicine, Xianyang, Shaanxi, China; 2 Affiliated Hospital of Shaanxi University of Chinese Medicine, Xianyang, Shaanxi, China; 3 The Sixth Affiliated Hospital of Xinjiang Medical University, Urumqi, Xinjiang, China; 4 Xinjiang Uygur Autonomous Region Orthopedic Hospital, Urumqi, Xinjiang, China; 5 Shaanxi University of Chinese Medicine, Xi’an, Shaanxi, China

**Keywords:** immunometabolic reprogramming, inflammaging, mitochondrial dysfunction, osteoarthritis, osteochondral unit, osteoporosis

## Abstract

Osteoporosis (OP) and osteoarthritis (OA) are common and highly co-occurring degenerative bone diseases in the elderly population, and their comorbidity mechanisms cannot be fully explained by traditional factors such as mechanical load, changes in bone mass, or hormonal imbalance. OP is primarily characterized by systemic decrease in bone mass and reduced bone strength, while OA is mainly manifested by cartilage degeneration within specific joints, synovial inflammation, and abnormal subchondral bone remodeling. Although both are influenced by aging, inflammation, and metabolic abnormalities, there are significant differences in their affected areas and pathological processes. In recent years, increasing evidence suggests that immunosenescence, chronic low-grade inflammation, and structural-functional imbalance of the osteochondral unit may constitute an important pathological basis for the progression of both diseases. Immunometabolic reprogramming is gradually being recognized as a potential mechanistic framework linking bone loss and joint degeneration, involving multiple processes such as enhanced glycolysis, impaired oxidative phosphorylation, lipid metabolism disorders, dysregulation of the NAD^+^/Sirtuins axis, and mitochondrial dysfunction, which can affect the functional status of key cell types including osteoclasts, osteoblasts, chondrocytes, and synovial macrophages. However, current evidence on the involvement of immunometabolic reprogramming in the progression of OP-OA comorbidity primarily comes from single-disease studies of OP or OA, while direct evidence from OP-OA comorbidity cohorts, integrated osteochondral unit models, and longitudinal clinical studies remains limited. Therefore, existing research is insufficient to establish that immunometabolic reprogramming constitutes a definitive causal pathway for the development of OP-OA comorbidity; rather, it should be regarded as a candidate integrative mechanism and a hypothesis to be validated for explaining the shared pathological basis of the two conditions. Centered on the thematic axis of “immunosenescence–immunometabolic reprogramming–osteochondral unit degeneration,” this article systematically reviews the key mechanisms, potential therapeutic insights, and current translational challenges in the comorbidity of osteoporosis and osteoarthritis, aiming to summarize the existing evidence base, clarify mechanistic boundaries, and provide new theoretical references for mechanistic analysis, risk stratification, and targeted intervention in OP-OA comorbidity.

## 1 Introduction

Osteoporosis (OP) and osteoarthritis (OA) are the two most common degenerative bone diseases in the elderly population, characterized by bone loss, destruction of bone microarchitecture, and articular cartilage degeneration, synovial inflammation, and subchondral bone remodeling, respectively (1). With the aging of the population, the coexistence of OP and OA in the same patient has attracted increasing attention, but the comorbidity rate varies depending on the study population, diagnostic criteria, affected sites, and evaluation methods (2, 3). The comorbidity of OP and OA not only increases the risk of pain, limited mobility, accidental falls, and fractures but also significantly exacerbates functional impairment and the medical burden (4). Therefore, elucidating the common pathological basis of their comorbidity is of great significance for risk stratification and comprehensive intervention of degenerative bone and joint diseases in the elderly.

Traditional views often attribute osteoporosis to an imbalance in bone remodeling, estrogen deficiency, and increased bone resorption, while osteoarthritis is considered the result of abnormal mechanical loading, cartilage degeneration, and abnormal subchondral bone remodeling (5). However, OP and OA are not simple manifestations within the same disease spectrum but are disease entities with different pathological scales and tissue characteristics: OP reflects systemic bone metabolic abnormalities and decreased bone strength, whereas OA primarily manifests as local joint structural degeneration and dysfunction. Despite their significant differences, traditional mechanisms are still insufficient to fully explain their frequent co-occurrence and mutual influence in the elderly population. In recent years, an increasing number of studies have suggested that inflammaging, immune imbalance, oxidative stress, mitochondrial dysfunction, and metabolic abnormalities may constitute an important pathological background for their common progression (6, 7). Among these, inflammaging, as a key biological feature of aging, can provide a new perspective for understanding the pathological link between the two by affecting bone remodeling, cartilage homeostasis, and the local immune microenvironment (8). The osteochondral unit, composed of articular cartilage, calcified cartilage, and subchondral bone, is an important structural basis for maintaining joint mechanical transmission and tissue homeostasis (9). Recent studies have found that bone and joint tissues are not independent pathological units but form a dynamic interaction network through cell communication, immune regulation, and changes in metabolic status (10). Therefore, bone loss and joint degeneration may not occur completely independently but influence each other against the background of common microenvironmental changes and cellular functional imbalances.

Immune metabolic reprogramming provides a new theoretical framework for understanding OP-OA comorbidity. The metabolic status of immune cells and osteochondral-related cells can affect their functional phenotypes and participate in bone remodeling, inflammatory regulation, and tissue repair processes (11). In recent years, alterations in glycolysis, oxidative phosphorylation, lipid metabolism, and NAD^+^-related metabolic regulation have been implicated in bone metabolic imbalance in OP and in cartilage and synovial pathology in OA, respectively (12, 13). However, current evidence mainly comes from studies focusing on OP or OA in isolation, and there is still insufficient evidence to establish that these form a common causal regulatory network in human OP-OA comorbidity. Therefore, immune metabolic reprogramming is more suitable as a candidate integrative framework for explaining the links among inflammaging, bone metabolic abnormalities, and osteochondral unit imbalance, rather than an established comorbidity-driving mechanism.

Unlike previous reviews that separately discussed the inflammatory mechanisms of OP or OA, this article, from the perspective of OP-OA comorbidity, explores the potential links among inflammaging, immune metabolic reprogramming, and osteochondral unit imbalance. It focuses on analyzing the shared pathological basis, changes in the immune microenvironment, key metabolic regulatory mechanisms, and related intervention strategies. At the same time, based on current research progress, this article discusses the main challenges in OP-OA comorbidity research, including the lack of unified disease definitions and classification criteria, insufficient direct comorbidity models and population evidence, the predominance of mechanistic studies derived from single-disease research, and limited clinical translational evidence, aiming to provide a new theoretical basis for the analysis of OP-OA comorbidity mechanisms and the development of precise intervention strategies.

## 2 Pathological basis of the comorbidity of osteoporosis and osteoarthritis

### 2.1 Physiological bone formation, bone remodeling, and osteochondral unit homeostasis

Normal bone tissue is not a static structure but maintains bone mass, microstructure, and mechanical integrity through continuous bone remodeling. Bone remodeling comprises a sequential cycle of osteoclast-mediated bone resorption, reversal, and osteoblast-mediated new bone formation and mineralization, during which bone resorption and formation maintain dynamic balance in time and space (14). Osteoclasts are responsible for removing aged or damaged bone matrix, followed by osteoblast recruitment and secretion of type I collagen and other bone matrix components to complete new bone formation and mineralization (15). Meanwhile, osteocytes embedded in the bone matrix act as important mechanosensory cells, sensing local mechanical changes and regulating signals such as receptor activator of nuclear factor-κB ligand/osteoprotegerin (RANKL/OPG) and Wnt/β-catenin to coordinate bone resorption and formation (16). Therefore, bone remodeling is a dynamic process involving multiple bone cell types and regulated by the local microenvironment.

Bone remodeling is also influenced by the immune microenvironment and systemic metabolic status. Interactions among osteoblasts, osteoclasts, osteocytes, and immune cells in the bone marrow microenvironment through cell contact, paracrine signaling, and cytokine networks collectively maintain bone tissue homeostasis (17). As aging progresses or when hormonal changes, chronic inflammation, and metabolic abnormalities persist, the functional coordination among bone cells becomes progressively compromised, leading to an imbalance between bone formation and resorption and promoting the development of bone metabolic diseases (18).

This homeostasis extends to the articular osteochondral unit. Although articular cartilage, calcified cartilage, and subchondral bone have different tissue structures and functional characteristics, they establish an intimate structural and functional coupling through mechanical transmission, nutrient exchange, and cellular signaling (19). Subchondral bone not only provides mechanical support for the overlying cartilage but also participates in local tissue signal regulation; cartilage maintains joint function stability by absorbing and dispersing mechanical loads (20). Thus, under normal conditions, bone remodeling and the osteochondral unit remain coordinated; however, with aging and the accumulation of disease factors, this homeostasis may gradually be disrupted, manifesting as abnormal bone remodeling in OP and structural degeneration of joints in OA.

### 2.2 Osteoporosis: imbalance of bone remodeling, bone loss, and pathological boundaries with osteoarthritis

OP is a systemic metabolic bone disease characterized by reduced bone mass, deterioration of bone microstructure, and decreased bone strength (21). The fundamental pathophysiological mechanism involves an imbalance between resorption and formation during bone remodeling, where osteoclast-mediated bone resorption exceeds osteoblast-mediated bone formation, leading to continuous bone loss and weakened mechanical properties (22, 23). As the disease progresses, structural changes such as reduced trabecular bone number, decreased connectivity, and cortical thinning further increase the risk of fragility fractures (24).

The occurrence of OP is influenced by multiple factors, including aging, estrogen deficiency, genetic factors, altered mechanical loading, and chronic diseases (25, 26). Throughout disease progression, enhanced osteoclast activity and decreased osteoblast function lead to abnormal coupling of bone remodeling, resulting in increased bone resorption and insufficient bone formation, ultimately causing bone loss and structural degeneration (27, 28). Abnormal bone turnover markers, such as the bone formation marker P1NP and the bone resorption marker β-isomerized C-terminal telopeptide of type I collagen, also reflect the disruption of the dynamic balance of bone metabolism (29, 30). OP is characterized not only by decreased bone mineral density (BMD) but also reflects overall abnormalities in bone tissue structure, cellular function, and the bone metabolic environment. However, although both OP and OA are common in the elderly population and are influenced by factors such as aging and metabolic abnormalities, they have distinct disease attributes and pathological scales.

OP primarily manifests as systemic bone loss and reduced bone strength, with clinical diagnosis mainly based on BMD measured by dual-energy X-ray absorptiometry (DXA), combined with a history of fragility fractures and fracture risk assessment (31). In contrast, OA is a chronic degenerative disease characterized by local structural degeneration of joints, primarily involving articular cartilage, synovium, and subchondral bone (32). Therefore, the potential link between OP and OA does not arise from the same bone phenotype but may be established on a common background of aging, disruption of tissue homeostasis, and abnormalities in multi-system regulation.

### 2.3 Osteoarthritis: cartilage degeneration, synovial inflammation, and subchondral bone remodeling

OA is a chronic degenerative disease affecting the entire joint organ, with pathological changes including progressive destruction of articular cartilage, synovial inflammation, subchondral bone remodeling, osteophyte formation, and pain and dysfunction (33, 34). Unlike the stable structural and functional coupling between cartilage and subchondral bone under normal conditions, during OA development, the coordination among cartilage matrix homeostasis, synovial immune environment, and subchondral bone remodeling gradually deteriorates (35). Chondrocytes are the core cells maintaining cartilage matrix homeostasis; under disease conditions, their anabolic capacity decreases, while the expression of matrix-degrading molecules such as matrix metalloproteinases (MMPs) and a disintegrin and metalloproteinase with thrombospondin motifs (ADAMTs) increases, leading to the degradation of type II collagen and proteoglycans, promoting cartilage degeneration (36).

Synovial inflammation is an important driving factor in OA progression, rather than a mere secondary event to cartilage damage (37). Macrophages, T cells, and synovial fibroblasts in OA synovial tissue are persistently activated, thereby releasing IL-1β, TNF-α, IL-6, prostaglandin E2 (PGE2), and various matrix-degrading factors (38). These inflammatory mediators not only promote cartilage matrix destruction but can also alter the local joint immune environment, creating a sustained inflammatory amplification process.

Subchondral bone remodeling is another key aspect of OA development and engages in a bidirectional crosstalk with cartilage degeneration. Under normal conditions, subchondral bone remodeling adapts to changes in joint loading to maintain appropriate mechanical support; however, following the onset of OA, this adaptive process gradually transitions into maladaptive remodeling (39). In early OA, enhanced bone resorption and changes in trabecular bone structure may occur in subchondral bone; as the disease progresses, abnormal osteogenesis, subchondral bone sclerosis, osteophyte formation, and abnormal vascularization gradually appear (40). These changes in subchondral bone not only affect the local mechanical environment but can also further promote cartilage degeneration by altering inter-tissue signaling (41). Therefore, cartilage degeneration, synovial inflammation, and abnormal subchondral bone remodeling in OA are not independent events but collectively constitute the disease progression process, providing an important basis for understanding the potential link between OA and OP.

### 2.4 Osteochondral unit: structural basis connecting OP and OA

The osteochondral unit, composed of articular cartilage, calcified cartilage, subchondral bone plate, and subchondral trabecular bone, is an important structural entity maintaining joint mechanical transmission, nutrient exchange, and tissue homeostasis (42). Under normal conditions, dynamic balance is maintained between cartilage and subchondral bone through mechanical adaptation and biological signaling. However, with the cumulative effects of aging, chronic inflammation, and metabolic abnormalities, this stable coupling is gradually disrupted, leading to cartilage degeneration, abnormal subchondral bone remodeling, and loss of local tissue homeostasis (43).

During OA development, subchondral bone is not merely a passively affected tissue but an important regulatory unit involved in the disease process (44). Early OA may exhibit enhanced subchondral bone resorption and microstructural changes, which may weaken its mechanical support and further increase abnormal loads on cartilage, accelerating cartilage degeneration (45, 46). Some animal studies suggest that low bone mass may affect the structural stability of subchondral bone and alter the progression of OA, but direct causal relationships in human populations still require further validation (47).

It is particularly important to emphasize that OP-related bone loss and OA-related subchondral bone remodeling are not the same pathological process. OP primarily reflects a systemic imbalance in bone remodeling, with core manifestations of reduced bone mass and increased fracture risk; in contrast, OA-related bone changes are mainly confined to the affected joint, manifesting as phased bone resorption, abnormal osteogenesis, and bone sclerosis (48, 49). Therefore, the “osteoporosis-like” subchondral bone changes in early OA do not equate to systemic osteoporosis but rather reflect abnormal remodeling in the local joint microenvironment. Similarly, subchondral bone sclerosis in late-stage OA does not represent an improvement in bone mass status but is the result of long-term mechanical stimulation and abnormal bone formation.

In turn, cartilage degeneration can reciprocally affect subchondral bone status. Cartilage matrix degradation products can be recognized by local immune cells, further influencing the synovial and subchondral bone microenvironment and promoting disease progression (50, 51). This bidirectional regulation between cartilage and subchondral bone suggests that the osteochondral unit is an important structural basis connecting local joint pathology with systemic bone metabolic status.

Overall, although OP and OA manifest as different levels of skeletal degeneration, they may be influenced by common aging factors, disruptions in tissue homeostasis, and abnormalities in multi-system regulation. The osteochondral unit, as an important platform connecting systemic bone metabolic status with local joint degeneration, provides a theoretical basis for further exploring the inflammatory, immune, and metabolic mechanisms in the OP–OA comorbidity (**Figure 1**).

**Figure 1:**
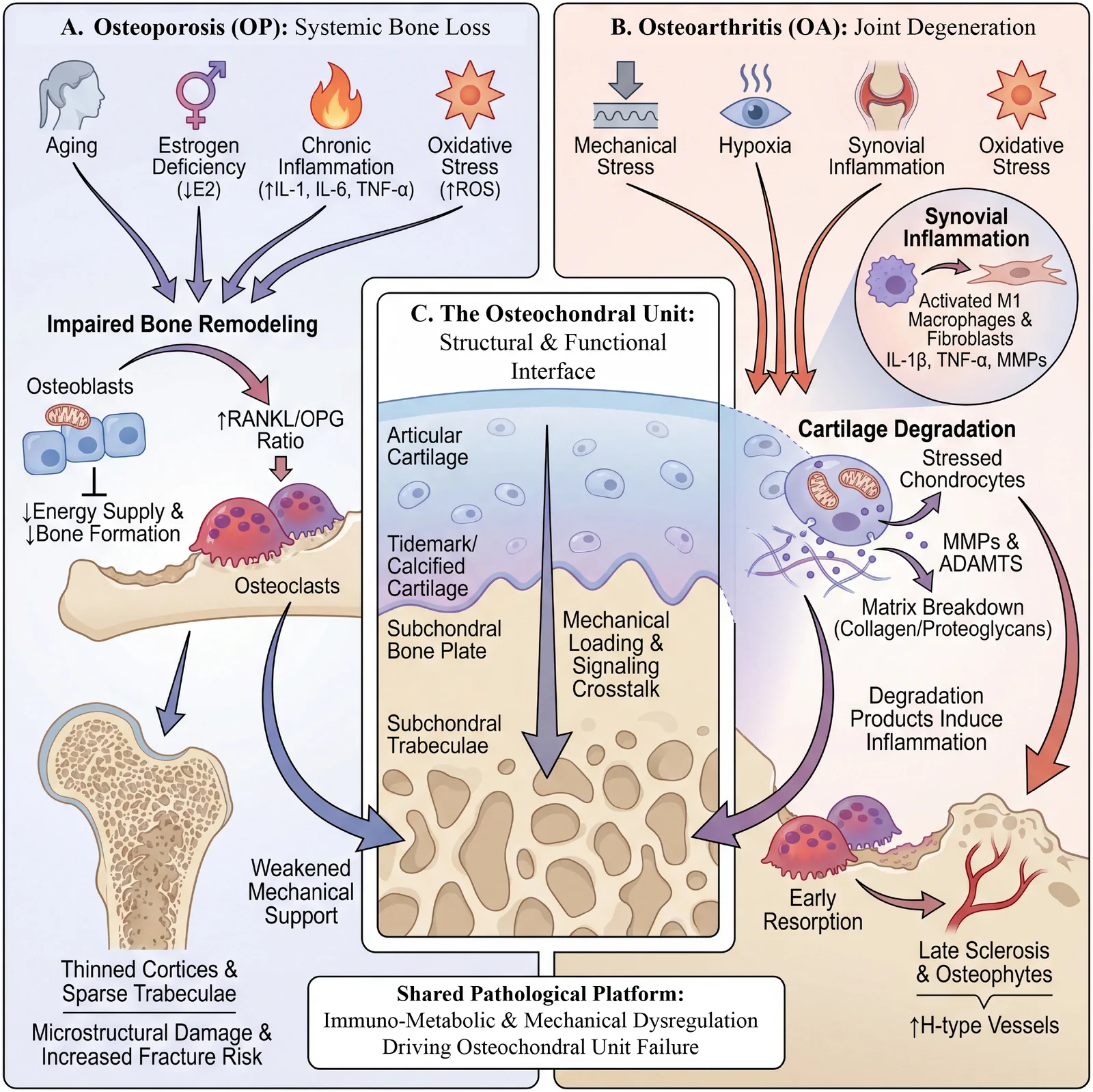
Common pathological basis of osteochondral unit dysfunction in osteoporosis and osteoarthritis. This figure outlines how osteoporosis and osteoarthritis jointly affect the osteochondral unit through immuno-metabolic and mechanical signal imbalance. **(A)** In osteoporosis, aging, estrogen deficiency, chronic inflammation, and oxidative stress lead to inhibited osteogenesis, enhanced osteoclast activity, and RANKL/OPG imbalance, resulting in bone loss and increased fracture risk. **(B)** In osteoarthritis, mechanical stress, hypoxia, synovial inflammation, and oxidative stress promote chondrocyte damage, upregulation of MMPs/ADAMTS, matrix degradation, and subchondral bone remodeling. **(C)** Articular cartilage, calcified cartilage, and subchondral bone together constitute the osteochondral unit, whose structural and functional imbalance is an important pathological platform for the cross-progression of OP and OA. This image was drawn using Adobe Illustrator software.

## 3 Inflammaging: the common pathological background of OP-OA comorbidity

### 3.1 Age-related chronic low-grade inflammation

During the aging process, the immune system gradually deviates from homeostasis, forming a persistent, low-grade pro-inflammatory state known as inflammaging. Its typical features include mildly but persistently elevated levels of circulating inflammatory markers such as IL-6, TNF-α, and C-reactive protein (52). Epidemiological and basic research suggests that inflammaging is closely associated with decreased BMD, increased risk of Bone fractures, joint pain, and cartilage degeneration, serving as one of the common pathological backgrounds for age-related musculoskeletal diseases such as OP and OA (53).

Inflammaging can affect bone and joint homeostasis through multiple pathways. In bone tissue, pro-inflammatory cytokines such as IL-6, TNF-α, and IL-1β promote osteoclast differentiation and bone resorption while inhibiting osteoblast differentiation, mineralization, and bone formation, thereby exacerbating bone remodeling imbalance (54). In joint tissue, these inflammatory mediators induce enhanced catabolic activity in chondrocytes, promote the expression of matrix-degrading enzymes such as MMPs and ADAMTS, and aggravate synovial inflammation and abnormal subchondral bone remodeling (55). Therefore, inflammaging may contribute to the co-progression of OP and OA by altering the local microenvironment of bone, cartilage, synovium, and subchondral bone, and is considered a key contributing factor for the shared pathological basis of the two conditions.

The sources of inflammaging are multifactorial, primarily including the accumulation of senescent cells, release of damage-associated molecular patterns (DAMPs), mitochondrial dysfunction, and Gut microbiota dysbiosis (56). With aging, senescent cells progressively accumulate in tissues such as bone marrow, cartilage, and synovium, and continuously release pro-inflammatory cytokines, chemokines, and matrix-remodeling enzymes through the senescence-associated secretory phenotype (SASP) (57). Meanwhile, DAMPs released by damaged cells are recognized by pattern recognition receptors, activating innate immune responses (58). Additionally, age-related Gut microbiota imbalance and decreased intestinal barrier function may increase the exposure of microbial-associated molecules such as lipopolysaccharide (LPS) to the host immune system, while dietary patterns, nutritional status, multimorbidity, and long-term medication exposure can further influence this microecological environment (59, 60). These systemic and local factors collectively maintain the activation of inflammatory signaling pathways such as NF-κB and JAK-STAT, forming a chronic inflammatory microenvironment conducive to bone loss and joint degeneration.

### 3.2 Gut microbiome–immune–bone/joint axis: age-related immunometabolic regulation

The gut microbiome undergoes dynamic changes with age, and its composition and function are simultaneously influenced by dietary patterns, underlying diseases, and medication exposure (61). Older adults and aging populations often experience reduced dietary fiber intake, altered nutritional patterns, multimorbidity, and long-term medication use, all of which may affect microbial diversity and metabolic function (62, 63). Chronic use of medications such as antibiotics, proton pump inhibitors, and certain metabolic drugs can further alter microecological composition and contribute to the pathogenesis and progression of bone and joint diseases by affecting host immune homeostasis and metabolic status (64). Therefore, when exploring the relationship between the gut microbiome and OP-OA comorbidity, it is necessary to fully consider the microecological heterogeneity caused by age-related lifestyle and medical factors.

Gut-derived signals can further influence the bone and joint immune environment. Microbial-associated molecules and their metabolites can regulate the function of monocytes/macrophages and T cells, and affect osteoclastogenesis, synovial inflammation, and cartilage catabolism through inflammatory mediators such as IL-1β, IL-6, TNF-α, and IL-17 (65). Among these, the Th17/regulatory T cells (Treg) balance may be a key link connecting the gut immune environment and bone metabolism, but current evidence is mainly derived from single-disease studies of OP or OA and inflammatory disease models, and its specific role in OP-OA comorbidity requires further validation (66).

Microbiota-derived metabolites are important mediators between the gut and bone/joint tissues. Short-chain fatty acids (SCFAs) can participate in intestinal barrier maintenance, Treg differentiation, macrophage function, and osteoclast formation (67, 68); secondary bile acids can regulate host metabolism and inflammation through FXR and TGR5, while tryptophan-derived indole metabolites can influence mucosal immunity and the Th17/Treg balance through the aryl hydrocarbon receptor (AhR) (69). However, the effects of these metabolites are highly dependent on cell type, tissue environment, and disease stage, and their role in OP-OA comorbidity is not simply protective or detrimental.

In addition to immune regulation, the gut microbiome may also affect bone homeostasis through hormone metabolism and mineral utilization. The estrobolome represents the functional network of gut microorganisms involved in estrogen metabolism, where bacterial-derived β-glucuronidase can influence the deconjugation of conjugated estrogens, regulating the enterohepatic circulation of estrogens and systemic estrogen levels (70). This process may affect the RANKL/OPG balance, osteoclast activity, and bone remodeling in postmenopausal women, while changes in estrogen levels may in turn regulate gut microbiome composition, forming a microbiome–hormone–immune regulatory network (71, 72). Furthermore, SCFAs and organic acids produced by microbial fermentation can alter the intestinal lumen environment, promoting the dissolution and absorption of minerals such as calcium, phosphorus, and magnesium, while intestinal barrier damage and microbial imbalance may further affect nutrient utilization efficiency, thereby influencing bone formation and bone turnover (73).

Overall, the gut microbiome may participate in OP-OA-related bone and joint homeostasis through multi-level mechanisms including immune regulation, microbiota-derived metabolites, estrogen metabolism, and mineral utilization. These mechanisms suggest that the gut microbiome may be an important regulatory factor connecting aging, immunometabolic abnormalities, and bone-cartilage unit imbalance. However, current evidence is still mainly derived from single-disease studies of OP or OA, and the microecological characteristics, mechanisms of action, and causal relationships in clinical cohorts of patients with OP-OA comorbidity still lack systematic validation.

### 3.3 SASP and immune cell senescence

SASP is an important mediator through which inflammaging affects the bone-cartilage unit. Senescent chondrocytes, osteoblasts, and bone marrow mesenchymal stem cells secrete SASP factors such as IL-1β, IL-6, IL-8, TNF-α, chemokines, and MMPs, altering the local immune and matrix microenvironment (74, 75). In bone tissue, the SASP of senescent osteoblasts and bone marrow mesenchymal stem cells can weaken osteogenic differentiation capacity and promote osteoclastogenesis through pro-inflammatory cytokines and RANKL-related signals, thereby exacerbating bone resorption and bone loss (76). In joint tissue, SASP factors released by senescent chondrocytes can induce the expression of matrix-degrading enzymes such as MMP13 and ADAMTS5, promote cartilage matrix degradation, and simultaneously affect synovial immune cell activation, aggravating local inflammatory responses (77).

Immunosenescence further influences the tissue microenvironment changes mediated by SASP. With aging, the function of immune cells such as T cells, macrophages, and NK cells gradually declines, manifested as reduced immune surveillance, imbalanced inflammatory regulation, and decreased ability to clear senescent cells (78). Among these, the decreased ability of macrophages to clear senescent cells may lead to the continuous accumulation of SASP source cells, while an increased proportion of pro-inflammatory macrophages can promote the release of inflammatory mediators such as IL-1β, IL-6, and TNF-α, thereby affecting osteoclast activation, synovial inflammation, and cartilage matrix homeostasis (79). Therefore, in OP and OA comorbidity, the significance of SASP and immunosenescence lies not only in amplifying systemic inflammation but also in persistently altering the local microenvironment of the bone-cartilage unit.

T cell senescence and Th17/Treg imbalance are considered potential immune mechanisms through which inflammaging affects bone-cartilage homeostasis (80). In aging and chronic inflammatory environments, Th17 cell activity is enhanced, while the function of immunoregulatory Treg cells may decline, leading to elevated levels of IL-17, RANKL, and other inflammatory factors (81). IL-17 can promote osteoclastogenesis and induce chondrocytes and synovial cells to produce inflammatory mediators and matrix-degrading enzymes, potentially participating in enhanced bone resorption and cartilage degeneration (82).

Current evidence regarding SASP, immunosenescence, and Th17/Treg imbalance is mainly derived from single-disease studies of OP or OA, as well as some chronic inflammatory disease models. Although these studies suggest that changes in immune cell function and abnormal inflammatory regulation may be involved in bone loss and joint degeneration, their specific mode of action and causal relationship in human OP-OA comorbidity have not been directly validated. Overall, SASP accumulation, decreased immune clearance capacity, and T cell functional imbalance may collectively shape the bone-cartilage microenvironment in the context of inflammaging, providing a potential basis for subsequent immunometabolic reprogramming; however, these mechanisms should currently be regarded as candidate regulatory factors for OP-OA comorbidity rather than established common pathogenic pathways.

### 3.4 Inflammaging-induced changes in the bone-cartilage microenvironment

Inflammaging can alter the local microenvironment of bone and joints through persistent low-grade inflammatory stimulation, affecting bone remodeling and bone-cartilage homeostasis. Pro-inflammatory cytokines such as IL-1β, TNF-α, and IL-6 can activate inflammatory signaling pathways such as NF-κB and MAPK, promoting osteoclast differentiation and bone resorption in bone tissue while inhibiting osteoblast differentiation and bone formation (83, 84). In joint tissue, persistent inflammatory stimulation can induce enhanced catabolic activity in chondrocytes, promote the expression of matrix-degrading enzymes such as MMP13 and ADAMTS5, and aggravate synovial inflammation and cartilage matrix destruction (85). Therefore, immune microenvironment changes mediated by inflammatory cytokines may be an important pathological link connecting bone loss and joint degeneration.

In addition to the direct effects of inflammatory cytokines, inflammaging can also persistently activate immune responses through DAMPs (86). DAMPs released by senescent or damaged cells are recognized by pattern recognition receptors, promoting the activation of macrophages, T cells, and other immune cells, and further enhancing the release of inflammatory cytokines, thereby establishing a self-perpetuating inflammatory amplification loop (87). In the bone-cartilage unit, this dysregulated immune activation can affect the interactions between osteoclasts, osteoblasts, chondrocytes, and synovial cells, promoting pathological bone remodeling and tissue degeneration.

Furthermore, inflammaging can affect the bone-cartilage microenvironment by altering local cell communication and immune clearance capacity. The accumulation of senescent cells, the continuous release of SASP, and decreased immune surveillance function cause pro-inflammatory cytokines, chemokines, and matrix-remodeling-related molecules to persist in the tissue environment, further promoting synovial inflammation, enhanced bone resorption, and disruption of cartilage homeostasis (88). Therefore, inflammaging is not only an important manifestation of systemic aging but also a key regulatory factor connecting immune imbalance and bone-cartilage unit abnormalities. It should be noted that changes related to inflammaging are closely associated with subsequent immunometabolic abnormalities; however, the primary role of inflammaging resides in forming a persistent inflammatory environment and altering cellular functional status. Specific mechanisms involving glycolysis reprogramming, mitochondrial dysfunction, and oxidative stress regulation will be further discussed in the subsequent immunometabolism section.

In summary, inflammaging provides a common pathological background for OP-OA comorbidity through the release of inflammatory cytokines, SASP accumulation, DAMP-mediated immune activation, and changes in the tissue microenvironment. However, current evidence is mainly derived from single-disease studies of OP or OA and experimental models, and the specific mode of action and causal relationship in human OP-OA comorbidity still require further validation (**Figure 2**).

**Figure 2:**
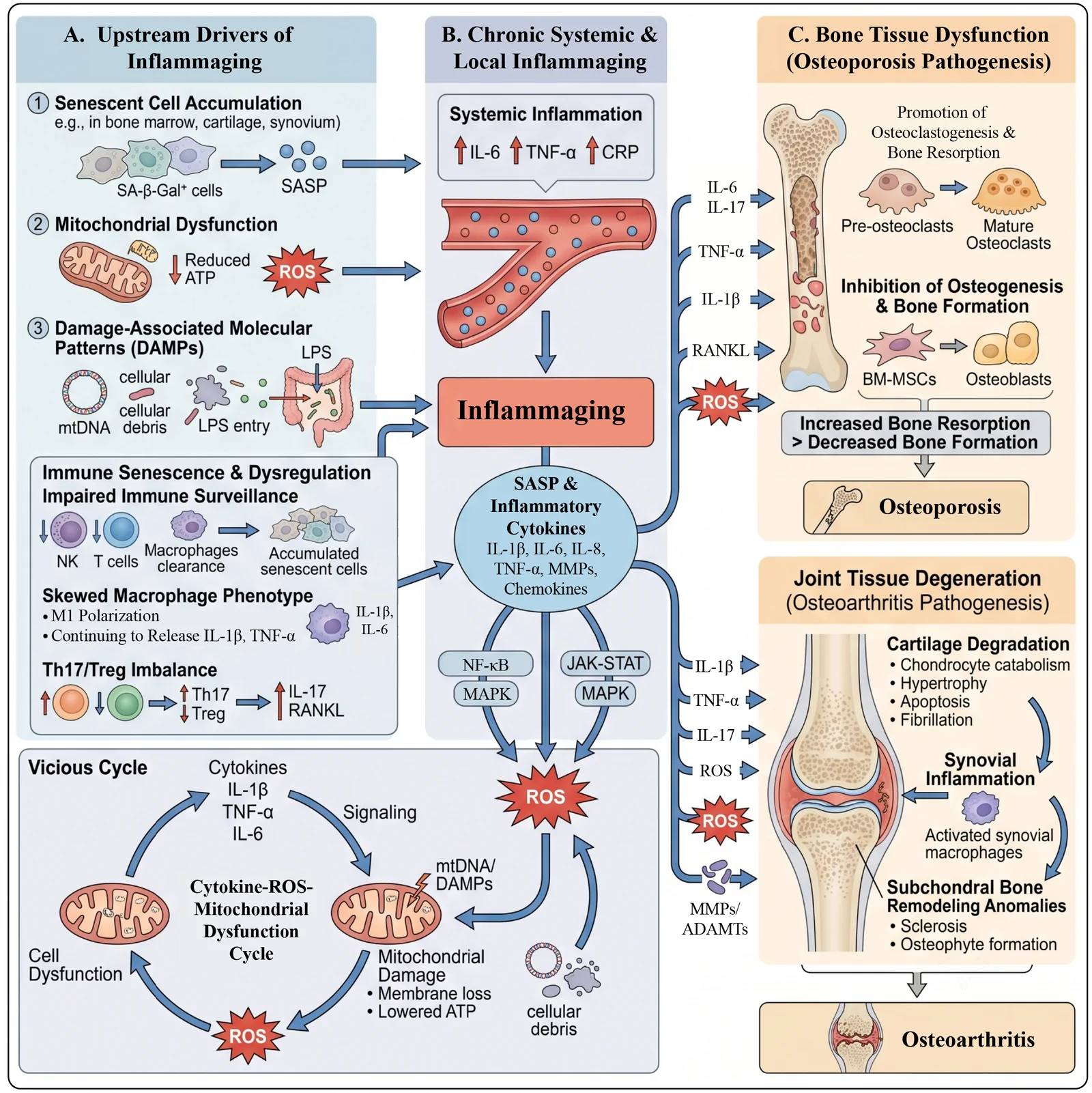
Common pathological mechanisms of inflammaging driving osteoporosis and osteoarthritis. This figure illustrates how senescent cell accumulation, mitochondrial dysfunction, DAMP/LPS release, and immunosenescence induce chronic systemic and local inflammation. **(A)** Upstream factors include SASP release, increased ROS, diminished immune clearance capacity, macrophage M1 polarization, and Th17/Treg imbalance. **(B)** Inflammatory mediators such as IL-1β, IL-6, TNF-α, IL-17, RANKL, and ROS amplify the inflammatory response through NF-κB, MAPK, and JAK-STAT pathways, forming a cytokine–ROS–mitochondrial damage vicious cycle. **(C)** In bone tissue, inflammaging promotes osteoclastogenesis and inhibits osteogenesis, leading to osteoporosis; in joint tissue, it promotes cartilage degradation, synovial inflammation, and subchondral bone remodeling, driving the progression of osteoarthritis. This image was drawn using Adobe Illustrator software.

## 4 Core mechanisms of immunometabolic reprogramming

It should be noted that current research evidence on immunometabolic reprogramming in OP–OA comorbidity exhibits significant heterogeneity. Based on the source of evidence, relevant studies can be categorized into three types: the first type is direct OP–OA evidence, primarily derived from comorbid clinical cohorts, animal models, or integrated studies of the osteochondral unit; the second type is indirect evidence from single diseases, mainly from independent OP or OA studies, which may suggest potential common mechanisms but are insufficient to establish their specific role in OP–OA comorbidity; the third type is extrapolated mechanistic evidence, mainly from models of aging, metabolic abnormalities, and inflammation-related diseases, used to provide mechanistic background and theoretical hypotheses. Therefore, the enhanced glycolysis, impaired oxidative phosphorylation, abnormal lipid metabolism, and NAD^+^/Sirtuins axis dysregulation discussed in this chapter should be understood as candidate immunometabolic mechanisms and testable hypotheses in OP–OA comorbidity, rather than confirmed common pathogenic pathways.

### 4.1 Enhanced glycolysis and pro-inflammatory immune phenotype

Current evidence on the involvement of glycolytic reprogramming in OP–OA-related pathological processes mainly comes from single-disease studies of OP or OA, while direct evidence from OP–OA comorbidity models remains scarce. Therefore, existing research primarily suggests that abnormal glycolysis may be a potential metabolic feature linking bone metabolic abnormalities and joint degeneration, rather than a confirmed common driving factor.

During immunometabolic reprogramming, activated immune cells, osteoclast precursors, and some tissue cells can exhibit enhanced glycolysis. Unlike cells in a resting state that primarily rely on oxidative phosphorylation (OXPHOS), activated cells can rapidly produce ATP and biosynthetic intermediates through aerobic glycolysis to meet the demands of proliferation, migration, and effector functions (89, 90).

Studies on lipopolysaccharide-stimulated macrophages show that immune cells can undergo a metabolic shift from OXPHOS to a Warburg-like metabolic pattern, characterized by increased glycolytic flux and a decreased proportion of mitochondrial oxidative metabolism (91). Meanwhile, glycolytic intermediates can enter the pentose phosphate pathway, providing substrates for nucleotide synthesis and reduced nicotinamide adenine dinucleotide phosphate (NADPH) generation, thereby maintaining cellular metabolic demands and redox balance (92). In bone metabolism, enhanced glycolysis supports the differentiation, fusion, and bone resorptive activity of osteoclast precursors (93); in joint tissues, metabolic alterations of chondrocytes may also affect their anabolic and catabolic balance (94). Thus, glycolytic reprogramming may serve as an important candidate metabolic node connecting abnormal bone resorption and cartilage degeneration.

Hypoxia-inducible factor 1 alpha (HIF-1α) is a key metabolic sensor regulating glycolytic adaptation. Under hypoxic or metabolic stress conditions, stabilization of HIF-1α promotes the expression of key glycolytic molecules such as glucose transporter 1 (GLUT1), hexokinase 2 (HK2), and lactate dehydrogenase A (LDHA), enhancing glycolytic flux (95). In osteoclasts, HIF-1α promotes the expression of osteoclast differentiation-related factors such as NFATc1, thereby enhancing bone resorption activity (96). In chondrocytes, inflammation and hypoxia-related metabolic reprogramming can also promote the expression of matrix-degrading enzymes such as MMPs and ADAMTS, driving cartilage matrix destruction (97). However, direct evidence is still lacking to establish that HIF-1α-mediated abnormal glycolysis serves as a common causal mechanism in OP–OA comorbidity.

Lactate is an important metabolic product of enhanced glycolysis and also a signaling molecule in immunometabolic regulation. Lactate can participate in cellular functional remodeling through G protein-coupled receptor 81 (GPR81), histone lactylation, and pyruvate kinase M2 (PKM2)-related regulation (98). It should be noted that lactate is not merely a pro-inflammatory metabolite; its effects depend on cell type, local concentration, and tissue environment. Under different conditions, lactate may either participate in tissue adaptation or influence chronic inflammation and tissue degeneration by altering cellular metabolic states. Therefore, in the context of OP–OA comorbidity, lactate is more suitable as a candidate metabolic signaling node connecting glycolysis, immune regulation, and changes in the osteochondral microenvironment.

### 4.2 Impaired oxidative phosphorylation and mitochondrial dysfunction

Mitochondrial dysfunction has been well-documented in both OP and OA studies, but it remains uncertain whether a common mitochondrial metabolic abnormality directly mediates OP–OA comorbidity. Thus, existing research primarily suggests that mitochondrial homeostasis imbalance may be a shared potential pathological basis for both conditions.

Impaired OXPHOS is an important manifestation of metabolic decline in bone and joint tissue cells. The mitochondrial electron transport chain is the core basis for ATP production, and its functional decline can lead to insufficient energy supply, increased ROS production, and enhanced cellular stress responses (99). In osteoporosis, osteoblast differentiation, matrix synthesis, and mineralization all depend on adequate energy supply. When aging, inflammation, or oxidative stress impairs the mitochondrial function of osteoblasts, decreased OXPHOS efficiency, reduced ATP production, and ROS accumulation can collectively inhibit osteogenic differentiation and mineralization, leading to insufficient bone formation (100). In osteoarthritis, although chondrocytes exist in a relatively hypoxic environment chronically, they still rely on mitochondria to maintain redox balance, ATP production, and cellular homeostasis. In OA chondrocytes, impaired mitochondrial complex function, decreased membrane potential, and insufficient ATP production can induce chondrocyte senescence, apoptosis, and increased expression of matrix-degrading enzymes, promoting cartilage degeneration (101).

Osteoclasts exhibit strong metabolic flexibility. Their differentiation and bone resorption functions not only depend on enhanced glycolysis but also require mitochondrial metabolism for cell fusion, formation of resorption lacunae, and energy supply (102). In different pathological stages, osteoclasts can dynamically switch between glycolysis and OXPHOS to adapt to inflammation, hypoxia, and changes in the bone microenvironment. Inflammatory factors and RANKL signaling can promote metabolic remodeling in osteoclast precursors, enhancing their capacity for differentiation and bone resorption (103). This metabolic flexibility makes osteoclasts an important cellular node connecting osteoporotic bone loss and early subchondral bone resorption in OA.

Mitochondrial quality control disorders further exacerbate OXPHOS damage. Under normal conditions, mitochondria maintain network stability through fusion, fission, and mitophagy, and promptly clear damaged mitochondria (104). However, in aging and inflammatory microenvironments, insufficient mitophagy can lead to the accumulation of damaged mitochondria, further increasing ROS production and reducing ATP generation (105). Excessive ROS can damage mitochondrial DNA, membrane structures, and respiratory chain proteins, forming a positive feedback loop of “mitochondrial damage—ROS accumulation—further mitochondrial damage.” Meanwhile, mtDNA and other mitochondria-derived DAMPs released from damaged mitochondria can activate innate immunity and inflammasome-related signals, further amplifying chronic inflammation within the osteochondral unit (106). Therefore, OXPHOS impairment, mitochondrial quality control abnormalities, and ROS-mediated inflammatory amplification may constitute an important candidate metabolic damage axis connecting OP and OA. However, it must be emphasized that this axis is currently primarily inferred from independent OP and OA studies, and its direct role in OP–OA comorbidity still lacks systematic validation.

### 4.3 Abnormal lipid metabolism, lactate accumulation, and NAD^+^/Sirtuins axis dysregulation

Similar to enhanced glycolysis and mitochondrial dysfunction, research evidence on lipid metabolism disorders and NAD^+^/Sirtuins axis abnormalities primarily comes from independent OP or OA studies. These metabolic changes may reflect common features in the context of aging, obesity, and chronic metabolic stress, but whether they constitute specific metabolic regulatory mechanisms in OP–OA comorbidity requires further validation.

Abnormal lipid metabolism is an important link connecting metabolic disorders, inflammatory responses, and osteochondral degeneration. Obesity, hyperlipidemia, and aging-related lipid metabolic abnormalities can lead to elevated free fatty acids (FFA), lipotoxicity accumulation, and increased bone marrow adiposity (107). Excessive FFA can activate inflammatory signals such as TLR4, inducing synovial macrophages to polarize toward a pro-inflammatory phenotype and promoting the release of inflammatory factors such as TNF-α, IL-1β, and IL-6 (108).

In bone tissue, bone marrow adiposity and lipotoxicity can inhibit osteoblast differentiation, promote osteoblast stress damage, and indirectly enhance osteoclastogenesis (109); in joint tissue, lipid metabolism disorders can exacerbate synovial inflammation, chondrocyte oxidative stress, and cartilage matrix degradation. Lipotoxicity can also induce endoplasmic reticulum stress and mitochondrial damage, further amplifying inflammation and metabolic disorders (110). Therefore, abnormal lipid metabolism not only reflects the background of metabolic comorbidities but may also participate in the common pathological processes of OP and OA. However, current evidence is insufficient to establish it as a definitive driving factor for the progression of both conditions.

In addition to lipid metabolism, NAD^+^/Sirtuins axis dysregulation is also an important manifestation of aging-related metabolic decline. With increasing age, decreased intracellular NAD^+^ levels can weaken the activity of NAD^+^-dependent deacetylases such as sirtuin 1 (SIRT1) and SIRT3, thereby affecting mitochondrial function, oxidative stress regulation, and inflammatory responses (111). Specifically, SIRT1 participates in maintaining mitochondrial homeostasis and regulating inflammation by modulating signaling pathways such as peroxisome proliferator-activated receptor gamma coactivator 1α (PGC-1α), NF-κB, and forkhead box O (FOXO); SIRT3 primarily maintains cellular metabolic stability by regulating mitochondrial protein deacetylation, respiratory chain function, and ROS clearance (112). Therefore, the decline of the NAD^+^/Sirtuins axis may serve as an important candidate mechanism connecting aging, metabolic functional decline, and tissue homeostasis disruption.

In OP-related studies, decreased NAD^+^/Sirtuins function is associated with osteoblast senescence, mitochondrial dysfunction, and reduced bone formation capacity; in OA studies, abnormalities in SIRT1/SIRT3 are linked to chondrocyte metabolic disorders, increased oxidative stress, and disruption of matrix homeostasis (113); additionally, metabolic stress and age-related changes may further affect the balance of NAD^+^ consumption and replenishment, forming a feedback regulatory network of “metabolic decline—mitochondrial dysfunction—cellular homeostasis abnormalities” (114). Thus, the NAD^+^/Sirtuins axis may be an important candidate regulatory pathway connecting aging, metabolic abnormalities, and osteochondral homeostasis imbalance. Although targeting NAD^+^ metabolism has certain mechanistic plausibility and preclinical research value, its true role and therapeutic potential in OP–OA comorbidity still require further validation through comorbidity models and clinical studies (**Figure 3**).

**Figure 3:**
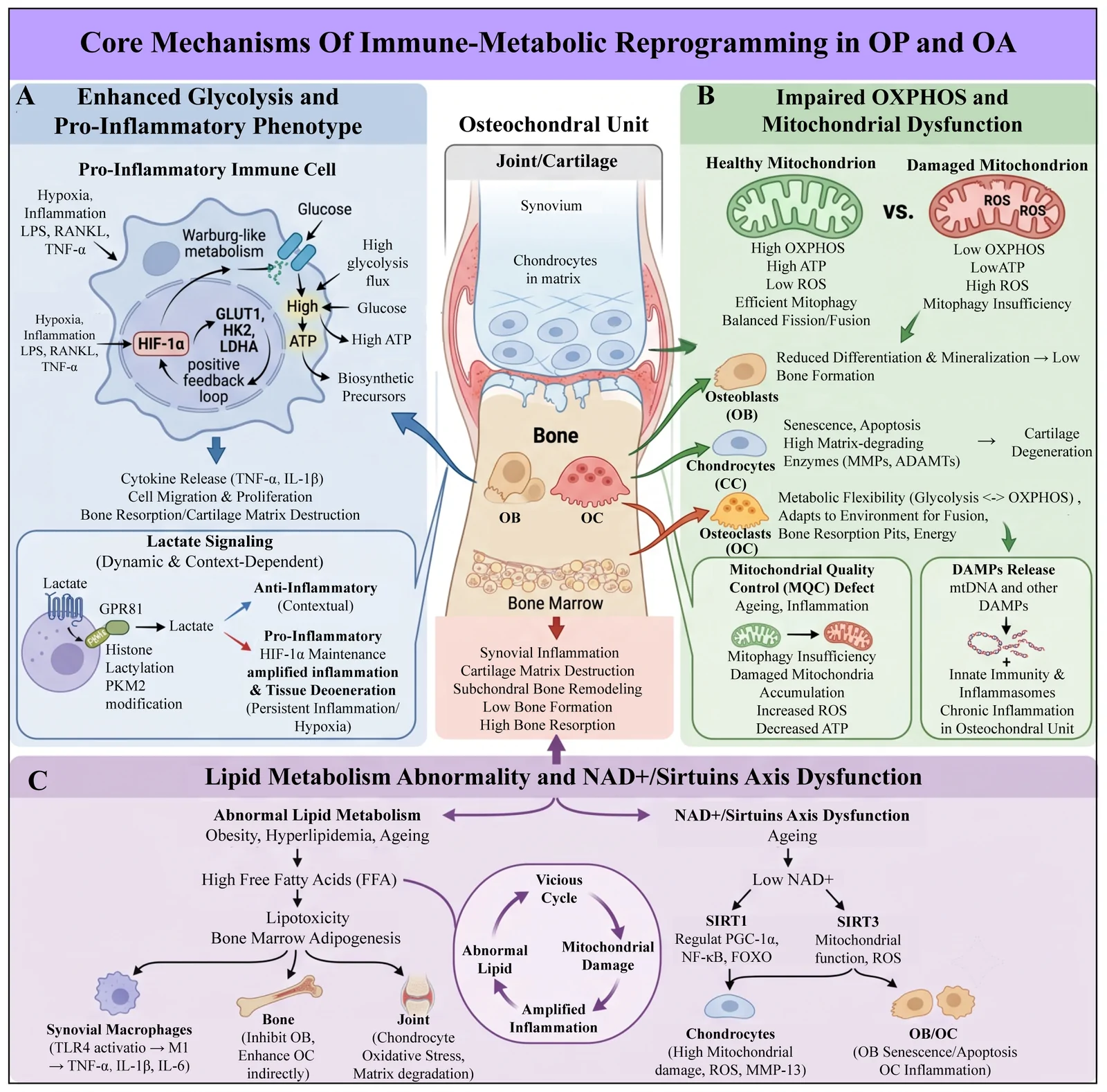
Core mechanisms of immunometabolic reprogramming in OP and OA. This figure outlines the key mechanisms underlying immunometabolic imbalance in the bone-cartilage unit in osteoporosis and osteoarthritis. **(A)** Stimuli such as hypoxia, inflammation, LPS, RANKL, and TNF-α promote enhanced glycolysis and HIF-1α positive feedback, inducing the release of pro-inflammatory cytokines, cell migration, and bone resorption and cartilage matrix destruction. **(B)** Impaired mitochondrial oxidative phosphorylation, elevated ROS, and defects in mitochondrial quality control can inhibit osteogenic differentiation and mineralization, promoting chondrocyte senescence, apoptosis, and matrix degradation. **(C)** Abnormal lipid metabolism and dysfunction of the NAD^+^/Sirtuins axis can further exacerbate synovial inflammation, decreased bone formation, osteoclast activation, and cartilage degeneration through lipotoxicity, mitochondrial damage, and amplification of inflammation. This image was drawn using Adobe Illustrator software.

Overall, enhanced glycolysis, impaired oxidative phosphorylation, abnormal lipid metabolism, and NAD^+^/Sirtuins axis dysregulation collectively constitute the currently proposed OP–OA immunometabolic candidate network. These metabolic changes have been supported in OP and OA studies respectively and may participate in abnormal bone remodeling and osteochondral unit imbalance by affecting the functions of macrophages, T cells, osteoclasts, osteoblasts, and chondrocytes. However, existing evidence is mainly derived from cell experiments, animal models, and single-disease studies, and cannot yet prove that the above metabolic pathways constitute common causal mechanisms in human OP-OA comorbidity. Future integration of comorbid population cohorts, single-cell multi-omics, spatial transcriptomics, and integrated osteochondral models will help further clarify the true pathological role of immunometabolic reprogramming in the development and progression of OP-OA comorbidity (**Table 1**).

**Table 1:** Evidence classification of immune metabolic reprogramming mechanisms potentially involved in OP–OA comorbidity.

Mechanism module	Key metabolic changes	Main cell/tissue effects	Immune inflammatory effects	Significance in OP–OA comorbidity	Evidence grade	Reference
Enhanced glycolysis	Activated immune cells shift from OXPHOS to Warburg-like glycolysis, with increased glucose uptake and glycolytic flux.	Pro-inflammatory macrophages, osteoclast precursors, and chondrocytes undergo metabolic activation.	Promotes cell migration, proliferation, release of inflammatory cytokines, and enhanced effector function.	Links inflammation amplification, osteoclast activation, and cartilage matrix destruction.	Animal + cell Indirect evidence for OP/OA single disease.	(89–92)
HIF-1α- mediated pro- inflammatory phenotype	Hypoxia, inflammation, and metabolic stress stabilize HIF-1α, upregulating glycolytic molecules such as GLUT1, HK2, and LDHA.	Macrophages maintain M1-like phenotype; osteoclast differentiation is enhanced; chondrocyte catabolism increases.	Promotes expression of inflammatory factors such as TNF-α, IL-1β, and MMPs/ADAMTS.	Drives increased bony resorption and cartilage degeneration, serving as a core node of glycolysis–inflammation coupling.	Animal + cell; Indirect evidence	(95–97)
Abnormal lactate signaling	Enhanced glycolysis leads to lactate accumulation, which regulates gene expression via GPR81, histone lysine lactylation, and PKM2 modification.	Affects macrophage polarization, osteoclast activation, and chondrocyte metabolic adaptation.	Exhibits bidirectional effects: it can participate in repair, but under sustained inflammation or hypoxia, it amplifies inflammation.	Acts as a dynamic signaling node connecting glycolysis, immune regulation, and local acidic microenvironment.	Cellular dominance; Limited animals; Mechanism extrapolation of evidence.	(98)
OXPHOS impairment	Decline in mitochondrial electron transport chain function, reduced ATP production, and increased ROS.	Osteoblast differentiation and mineralization are inhibited; chondrocyte senescence, apoptosis, and matrix degradation increase.	Energy deficiency and ROS accumulation jointly induce cellular stress and inflammatory responses.	Leads to insufficient bone formation in OP and cartilage degeneration in OA, forming the basis of bone–cartilage metabolic decline.	Human observation + animals + cells; Evidence of OP/OA single disease.	(99–101)
Osteoclast metabolic flexibility	Osteoclasts dynamically switch between glycolysis and OXPHOS to adapt to inflammatory and hypoxic microenvironments.	Promotes osteoclast differentiation, fusion, resorption lacuna formation, and bone resorption activity.	RANKL and inflammatory factors promote metabolic remodeling of osteoclast precursors.	Connects systemic bone loss in OP with early subchondral bone resorption in OA.	Animal + cell; Indirect comorbidity inference.	(102, 103)
Mitochondrial quality control impairment	Imbalance in mitochondrial fusion, fission, and mitophagy; insufficient clearance of damaged mitochondria.	Accumulation of damaged mitochondria, decreased ATP, and persistently elevated ROS.	Release of mtDNA and mitochondrial DAMPs activates innate immunity and inflammasome signaling.	Forms a “mitochondrial damage–ROS–inflammation amplification” cycle, exacerbating degeneration of the bone–cartilage unit.	Animal + cell; Indirect evidence.	(105, 106)
Abnormal lipid metabolis	Obesity, hyperlipidemias, and aging lead to elevated FFA levels, lipotoxicity, and bone marrow adiposity.	Inhibits osteoblast differentiation, promotes osteoblast stress; aggravates synovial inflammation and chondrocyte injury.	FFA can activate TLR4 and other inflammatory pathways, promoting M1-like macrophage polarization and release of inflammation cytokines.	Is an important driving factor for the co-progression of OP and OA in the context of metabolic syndrome.	Research on human correlation + animals + cells; Indirect evidence.	(107–110)
NAD^+^/Sirtuins axis dysregulation	Decline in NAD^+^ leads to reduced SIRT1/SIRT3 activity, decreased mitochondrial biogenesis and antioxidant capacity.	Increased osteoblast senescence/apoptosis; mitochondrial damage in chondrocytes and elevated MMP-13 expression.	Imbalance in NF-κB, FOXO, PGC-1α pathways, enhancing inflammation and oxidative stress.	Forms a vicious cycle of “lipid abnormality–NAD^+^ decline–mitochondrial damage–inflammation amplification”.	Animal + cell; Extrapolated evidence related to aging.	(111–114)

## 5 Immunometabolic reprogramming in osteoporosis

The immunometabolic abnormalities in OP are primarily characterized by functional changes in bone remodeling-related cells and the bone immune microenvironment. Current evidence on the immunometabolic mechanisms of OP primarily stems from osteoporosis models and clinical studies, while its association with OA is largely based on mechanistic insights into bone-cartilage unit homeostasis, subchondral bone remodeling, and shared metabolic abnormalities. Therefore, OP-related immunometabolic alterations not only reflect a systemic imbalance in bone metabolism but may also contribute to the OP-OA pathological crosstalk by influencing the subchondral bone microenvironment.

### 5.1 Metabolic remodeling of osteoclasts and enhanced bone resorption

Osteoclasts are the primary effector cells of bone resorption, and their differentiation, fusion, and resorptive function are highly dependent on metabolic remodeling. Upon stimulation by pro-osteoclastogenic signals such as RANKL and TNF-α, osteoclast precursors upregulate glucose uptake and glycolytic flux to meet the energy and biosynthetic demands required for mature osteoclast formation, cytoskeletal reorganization, and bone resorption (115, 116).

Studies have shown that RANKL-induced osteoclast differentiation is often accompanied by the upregulation of metabolic molecules such as GLUT1, HK2, LDHA, and pyruvate dehydrogenase kinase 4 (PDK4), thereby promoting ATP production, lactate generation, and acidification of the bone resorption microenvironment (117). These metabolic changes do not occur in isolation but are tightly coupled to functional processes including cell fusion, polarization, resorption lacuna formation, and maintenance of the acidified environment. Therefore, the metabolic state of osteoclasts should be understood as an important regulatory factor supporting their functional transition, rather than a result of abnormalities in a single metabolic pathway. Furthermore, osteoclasts possess considerable metabolic plasticity and can adjust energy sources and metabolic patterns according to local bone microenvironment, oxygen tension, and inflammatory status. At different disease stages, osteoclasts may maintain bone resorptive function through dynamic switching between glycolysis and mitochondrial metabolism (118, 119).

From the perspective of OP-OA comorbidity, osteoclast dysfunction may serve as a critical nexus between the two conditions. In OP, excessive osteoclast activation leads to systemic bone loss and destruction of bone microarchitecture; whereas in early OA, osteoclast activation and increased bone resorption can also occur in the subchondral bone region, further weakening the mechanical support of subchondral bone and promoting bone-cartilage unit imbalance (120). Therefore, osteoclast metabolic adaptation may link systemic bone loss and local subchondral bone remodeling. However, current evidence directly demonstrating that osteoclast metabolic alterations drive the development and progression of OP-OA comorbidity remains limited.

### 5.2 Energy metabolism dysfunction of osteoblasts and insufficient bone formation

Osteoblasts are the core functional cells of bone formation, and their differentiation, matrix synthesis, and mineralization processes all depend on adequate energy supply and stable mitochondrial function (121). During normal bone formation, osteoblasts require oxidative phosphorylation, mitochondrial biogenesis, and metabolic substrate switching to support collagen synthesis, matrix secretion, and mineralization (122). With aging and osteoporosis progression, the metabolic adaptive capacity of osteoblasts declines, manifesting as insufficient energy supply, disrupted mitochondrial homeostasis, and reduced osteogenic differentiation potential (123). Concurrently, the bone marrow microenvironment undergoes changes, wherein increased marrow adiposity alters mesenchymal stem cell lineage commitment, favoring adipogenic differentiation and reducing osteogenic potential (124, 125). Therefore, insufficient bone formation is not only related to decreased osteoblast number and activity but also closely associated with cellular metabolic status and local microenvironment remodeling.

Aberrations in lipid metabolism constitute a critical determinant of osteoblast dysfunction. Excessive lipid accumulation can alter the metabolic state of osteoblasts, induce cellular stress responses, and impair osteogenic differentiation capacity; simultaneously, increased lipid peroxidation and decreased antioxidant capacity may further promote ferroptosis-related changes, leading to reduced osteogenic marker expression and impaired bone matrix formation (126, 127). These findings suggest that bone marrow adiposity is not merely a structural tissue change but may affect osteoblast function by altering the immunometabolic microenvironment.

The Wnt/β-catenin signaling pathway is an important molecular network regulating osteoblast differentiation and bone formation. This pathway orchestrates the regulation of runt-related transcription factor 2 (RUNX2) expression, osteoblast maturation, and bone matrix mineralization (128, 129). In the osteoporotic state, metabolic stress, cellular stress, and local microenvironment changes can all affect Wnt/β-catenin signaling activity, thereby reducing osteoblast differentiation capacity and bone formation efficiency (130).

Overall, osteoblast dysfunction in OP is not caused by a single factor but is influenced by multiple factors including abnormal cellular metabolic adaptation, increased bone marrow adiposity, and imbalance in osteogenic regulatory networks. Notably, the aforementioned mechanisms are mainly derived from OP-related studies, while osteoblast dysfunction also participates in OA subchondral bone remodeling processes, including osteophyte formation and bone sclerosis. Therefore, osteoblast metabolic abnormalities may constitute a plausible mechanistic link linking insufficient bone formation in OP and local bone remodeling in OA, but their specific role in human OP-OA comorbidity warrants further investigation.

### 5.3 Macrophage- and T cell-mediated bone immune microenvironment imbalance

Macrophages and T cells in the bone immune microenvironment participate in bone remodeling regulation, inflammatory responses, and tissue repair, serving as important cell populations connecting immune status and bone metabolism (131, 132). Recent studies have shown that immune cell function is not determined by a single fixed phenotype but is influenced by multiple factors including tissue origin, disease stage, local microenvironment, and metabolic status (133). Therefore, although macrophage- and T cell-related mechanisms have been extensively studied in OP, OA, and other chronic inflammatory disease models, direct evidence regarding whether they constitute shared immune regulatory mechanisms in OP–OA comorbidity remains lacking.

Under conditions of aging, estrogen deficiency, and chronic inflammation, the bone marrow immune microenvironment can undergo remodeling, with corresponding changes in macrophage and T cell functional states associated with bone remodeling abnormalities (134, 135). The traditional M1/M2 classification originates primarily from functional descriptions under *in vitro* stimulation conditions and cannot fully reflect the physiological state of macrophages *in vivo*. In recent years, single-cell transcriptomic studies have identified multiple macrophage subpopulations with distinct functional characteristics in different tissues and disease environments, encompassing functions such as inflammatory regulation, phagocytic clearance, tissue repair, and metabolic adaptation (136).

In OP and OA-related studies, some macrophage subpopulations exhibit pro-inflammatory functional features resembling “M1-like” phenotypes, while others display characteristics related to tissue repair and inflammation resolution. These functional states are not mutually exclusive or fixed but can undergo dynamic transitions in response to local signals and disease stages (137). Different macrophage subpopulations can exhibit distinct metabolic adaptation patterns, and their functional changes may be influenced by glycolysis, lipid metabolism, and mitochondrial homeostasis regulation (138). From a tissue-specific perspective, bone marrow-derived macrophages participate in bone remodeling regulation and can maintain bone homeostasis by influencing bone-forming cells, osteoclasts, and the bone matrix environment (139); whereas synovial macrophages in OA joints exhibit more complex functional states, including inflammation amplification, inflammation resolution, and tissue repair processes (140). Therefore, macrophage dysregulation should not be oversimplified as “increased M1 causes disease and increased M2 promotes repair,” but rather viewed as a consequence of disrupted dynamic equilibrium among various functional states. Currently, the specific role of macrophage functional changes in human OP-OA comorbidity still requires further validation through more comorbidity models and clinical studies.

In addition to macrophages, T cell subset balance constitutes a critical aspect of bone immune regulation. Th17 and Treg cells participate in maintaining bone immune homeostasis by regulating inflammatory responses and bone resorption-related signals. Existing OP studies have shown that enhanced Th17 cell-related inflammatory responses may promote RANKL expression and affect osteoclast formation; whereas in OA and other inflammatory joint disease studies, IL-17 has been found to participate in synovial inflammation and cartilage matrix degradation (82, 141). Conversely, Treg cells limit excessive inflammatory responses through immunomodulatory effects and may participate in maintaining bone tissue homeostasis (142). However, it should be emphasized that current evidence on Th17/Treg-related mechanisms mainly derives from single-disease studies of OP, OA, and inflammatory disease models such as rheumatoid arthritis, and their specific role patterns and causal relationships in human OP-OA comorbidity have not been directly confirmed (143, 144). Therefore, these immune cell changes should currently be regarded as potential regulatory factors connecting inflammation, metabolic abnormalities, and bone-cartilage homeostasis imbalance, rather than definitively established comorbid pathogenic mechanisms.

Overall, macrophages and T cells may participate in OP-OA-related bone-cartilage microenvironment changes by influencing immune status, metabolic adaptation, and intercellular communication. Future studies combining OP-OA comorbidity patient cohorts, single-cell multi-omics, spatial transcriptomics, and integrated bone-cartilage models are expected to further clarify the true roles of different immune cell subpopulations in the development and progression of comorbidity (**Figure 4**).

**Figure 4:**
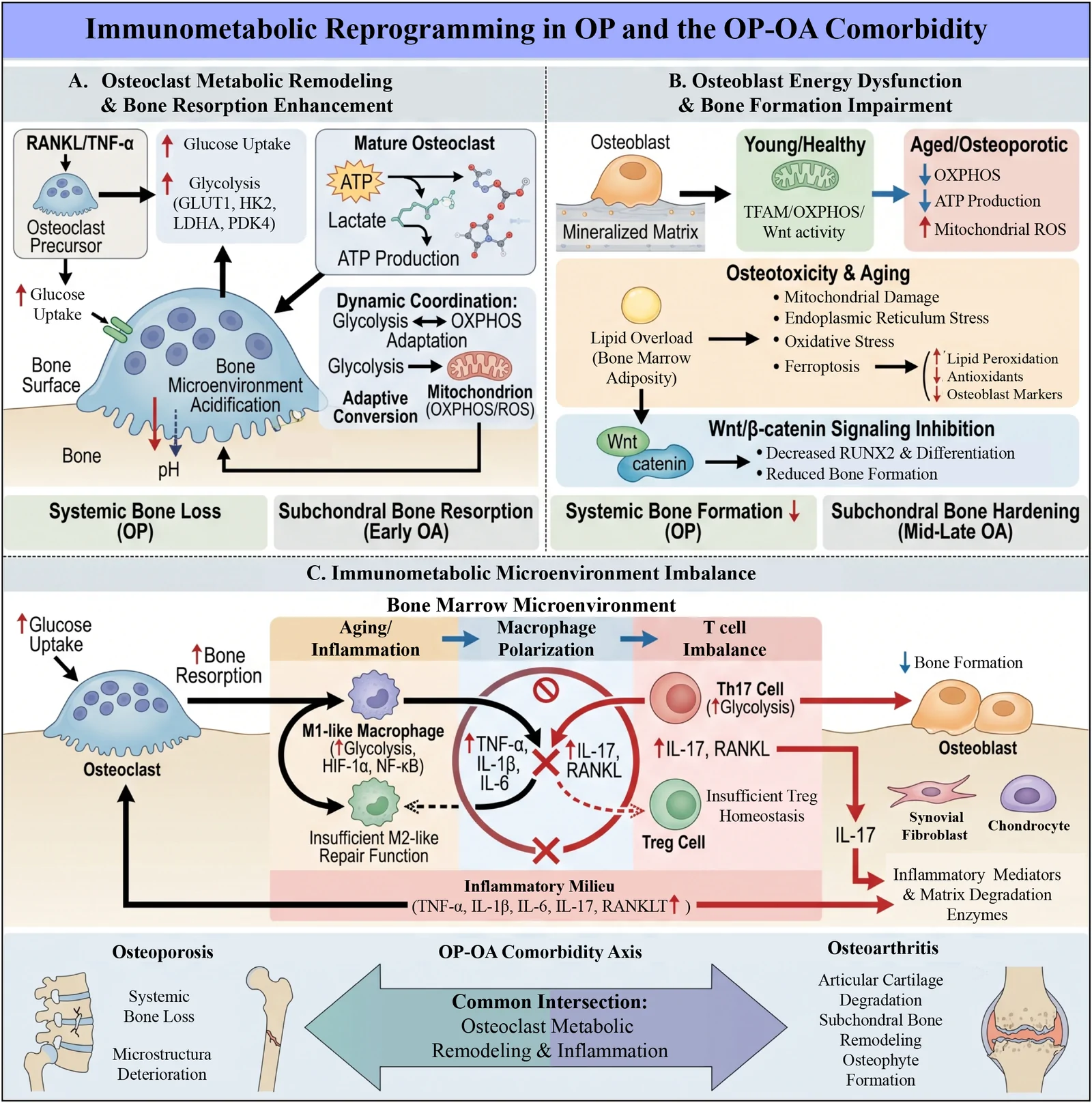
Immunometabolic reprogramming mechanisms in OP and OP–OA comorbidity. This figure outlines the bone metabolism and immune microenvironment imbalance in osteoporosis and osteoarthritis comorbidity. **(A)** RANKL/TNF-α promotes osteoclast glycolysis, lactate production, and formation of an acidic microenvironment, enhancing bony resorption and participating in systemic bone loss and early subchondral bony resorption. **(B)** Aging and osteotoxic stimuli lead to osteoblast mitochondrial dysfunction, decreased oxidative phosphorylation, increased ROS, and inhibition of Wnt/β-catenin signaling, resulting in reduced bone formation and subchondral bone sclerosis. **(C)** In the bone marrow microenvironment, M1-like macrophages, Th17/Treg imbalance, and elevated levels of TNF-α, IL-1β, IL-6, IL-17, and RANKL collectively amplify inflammation, osteoclast activation, osteoblast suppression, and cartilage matrix degradation, forming the OP–OA comorbidity axis. This image was drawn using Adobe Illustrator software.

## 6 Immunometabolic reprogramming in osteoarthritis

Abnormal immunometabolism in OA primarily involves impaired metabolic adaptation of chondrocytes, functional remodeling of synovial immune cells, and aberrant subchondral bone remodeling (145). Current evidence is mainly derived from OA patient samples and animal model studies, while its link to OP is inferred from the overall imbalance of the osteochondral unit, aging-related inflammation, and shared metabolic abnormalities. Therefore, OA-related immunometabolic changes may provide important clues for understanding OP-OA comorbidity, but more direct comorbidity studies are needed for validation.

### 6.1 Mitochondrial injury and matrix degradation in chondrocytes

Due to the lack of vascular, neural, and lymphatic supply in mature articular cartilage, chondrocytes exist in a long-term hypoxic, low-nutrient, and low-metabolic environment, making their energy metabolism highly sensitive to mitochondrial homeostasis, glycolytic adaptation, and redox balance (146). Thus, once inflammatory stimuli, mechanical overload, or aging factors disrupt the metabolic adaptability of chondrocytes, mitochondrial injury may become a key initiating factor in cartilage degeneration. In OA chondrocytes, mitochondrial DNA damage, decreased activity of electron transport chain complexes, reduced mitochondrial membrane potential, and insufficient ATP production often coexist with excessive ROS production (147, 148). This energy metabolic imbalance can activate inflammatory signaling pathways such as NF-κB and MAPK, promoting the upregulation of matrix-degrading enzymes like MMP13 and ADAMTS5, ultimately leading to the degradation of type II collagen and proteoglycans, thereby accelerating cartilage matrix destruction (149). Additionally, mechanical overload and aging factors can further exacerbate mitochondrial dysfunction, resulting in the accumulation of damaged mitochondria, elevated ROS, and enhanced catabolic activity of chondrocytes (150).

Abnormal mitochondrial dynamics and mitophagy are important mechanisms for the progressive propagation of mitochondrial dysfunction in chondrocytes. Under normal conditions, mitochondria maintain quality control through fusion, fission, and mitophagy, promptly removing dysfunctional mitochondria (151). However, in OA chondrocytes, impaired mitophagy can lead to the persistent accumulation of damaged mitochondria, further exacerbating ROS production and reducing ATP supply (152). For example, downregulation of Sirt4 can inhibit PTEN-induced kinase 1 (PINK1)-mediated mitophagy, leading to decreased mitochondrial membrane potential, ROS accumulation, and chondrocyte senescence; reduced LONP1 expression or abnormal fatty acid oxidation can also impair mitochondrial quality control, promoting MAPK signaling activation and increased MMP13 expression (153). In end-stage OA, a vicious cycle forms among insufficient mitophagy, mitochondrial injury, and chondrocyte apoptosis, resulting in decreased cartilage matrix synthesis and enhanced degradation (154). Since mitochondrial dysfunction also participates in osteoblast senescence and reduced bone formation capacity in OP, mitochondrial homeostasis imbalance may constitute an important aging-related metabolic mechanism linking OP and OA. However, this common mechanism is currently derived mainly from independent studies of the two diseases, lacking direct validation from OP-OA comorbidity models.

### 6.2 Glycolytic reprogramming of synovial macrophages and inflammatory amplification

Synovial inflammation is a key driver of OA progression, and metabolic reprogramming of synovial macrophages plays a significant role in this process. In OA, metabolic reprogramming of synovial macrophages typically acts in concert with cartilage matrix degradation products, DAMPs, and subchondral bone remodeling signals to maintain low-grade but persistent joint inflammation (140). Under continuous stimulation by DAMPs, hypoxia, and inflammatory factors, synovial macrophages can shift from a relatively homeostatic or reparative phenotype to an M1-like pro-inflammatory phenotype, characterized by enhanced glycolysis, HIF-1α stabilization, and decreased fatty acid oxidation (155). Enhanced glycolysis not only provides rapid energy and biosynthetic substrates for pro-inflammatory macrophages but also sustains their pro-inflammatory state through lactate accumulation, HIF-1α activation, and NF-κB signal amplification (156). Conversely, decreased fatty acid oxidation and oxidative phosphorylation may impair the capacity of macrophages to resolve inflammation and repair tissue, rendering the synovial microenvironment more susceptible to a persistent inflammatory state (138).

M1-like synovial macrophages after glycolytic reprogramming can secrete large amounts of pro-inflammatory cytokines such as IL-1β, TNF-α, and IL-6, activating synovial fibroblasts and chondrocytes in a paracrine manner (157). These inflammatory factors can induce the expression of matrix-degrading enzymes like MMPs and ADAMTS, directly promoting cartilage matrix destruction. Furthermore, they exert a feedback effect on the macrophages themselves, further activating NF-κB, MAPK, and HIF-1α-related signals, thereby forming a positive feedback loop of “enhanced glycolysis—pro-inflammatory cytokine release—synovial inflammation amplification—cartilage degradation” (158). The enhanced glycolysis of synovial macrophages in OA parallels the inflammatory activation of the bone marrow immune microenvironment in OP, suggesting that immune cell metabolic remodeling may be an important mechanism linking the two diseases. However, direct evidence confirming whether they share identical metabolic driving patterns remains lacking. Therefore, targeting the metabolic state of synovial macrophages, such as inhibiting abnormal glycolysis, restoring mitochondrial metabolic capacity, or promoting the transition from M1 to M2 phenotype, may become an important strategy for regulating the OA inflammatory microenvironment.

### 6.3 Subchondral bone remodeling and osteochondral unit imbalance

Subchondral bone remodeling is a crucial aspect of OA pathological progression and a key structural basis connecting cartilage degeneration, local bone metabolic abnormalities, and osteochondral unit imbalance. OA is not merely a cartilage disease but a comprehensive degenerative process involving articular cartilage, calcified cartilage, subchondral bone plate, and subchondral trabecular bone (159). During the early stages of the disease, subchondral bone typically exhibits active remodeling, accompanied by osteoclast activation, decreased bone volume fraction, trabecular thinning, and increased trabecular separation (160, 161). These local changes share some pathological similarities with enhanced bone resorption in OP, but they occur in different disease contexts: OP reflects systemic bone remodeling imbalance, while abnormal subchondral bone in OA mainly represents a local remodeling process within the affected joint. Therefore, they may share some inflammatory and immunometabolic regulatory mechanisms, but subchondral bone changes in OA cannot be simply regarded as a local manifestation of systemic osteoporosis.

In early OA, structural damage to the subchondral bone can weaken its mechanical support capacity, alter joint load distribution, and increase the mechanical stress borne by the overlying cartilage, thereby promoting cartilage matrix degradation and degeneration. Some animal studies have shown that low bone mass may exacerbate injury-induced or mechanical load-related OA phenotypes, suggesting that systemic bone metabolic abnormalities may participate in OA progression by affecting the stability of the osteochondral unit. However, this association is currently based mainly on animal models and mechanistic studies, yet sufficient longitudinal clinical evidence remains lacking regarding whether low bone mass directly influences the onset and progression of human OA.

As the disease progresses, OA subchondral bone remodeling gradually shifts from early bone resorption to abnormal osteogenesis and bone sclerosis. In the intermediate stage, increased vascular and neural invasion into the subchondral bone, along with changes such as H-type angiogenesis, platelet-derived growth factor-BB release, and local TGF-β abnormal activation, promote bone remodeling and further affect chondrocyte metabolism and phenotype stability (49). Mediators such as TGF-β not only drive abnormal osteogenesis of the subchondral bone but can also induce chondrocyte hypertrophy, increased MMP13 expression, and cartilage matrix degradation (162). Additionally, inflammatory cells and metabolic abnormalities can exacerbate early bone loss and subsequent abnormal remodeling of the subchondral bone by promoting osteoclast activation, the differentiation of myeloid-derived suppressor cells (MDSCs) into osteoclasts, the accumulation of lipotoxicity, and the release of inflammatory factors (163). This dynamic change indicates that the subchondral bone is not merely a supporting structure affected by mechanical factors but an active pathological microenvironment co-regulated by immune, metabolic, and local signals.

In late-stage OA, subchondral bone sclerosis, osteophyte formation, and abnormal mechanical conduction gradually become the main features. It is important to note that subchondral bone sclerosis in late-stage OA does not represent a reversal of the osteoporotic state but is the result of long-term mechanical stimulation, local inflammatory response, and abnormal bone formation. Unlike the systemic bone loss and fragile bone structure in OP, subchondral bone sclerosis in OA can be accompanied by increased bone formation, thickened trabeculae, and abnormal bone turnover, reflecting pathological adaptation and remodeling in the local joint environment. Subchondral bone sclerosis and osteophyte formation further alter the joint mechanical environment, aggravate cartilage damage, and disrupt the stability of the osteochondral unit (159).

Overall, OA subchondral bone remodeling exhibits a stage-specific pattern of “early increased bone resorption—intermediate abnormal osteogenesis and vascular/neural invasion—late bone sclerosis,” while OP is mainly characterized by persistent systemic bone loss. The connection between the two does not arise from the same bone phenotype but may intersect through common mechanisms such as inflammatory aging, immunometabolic abnormalities, oxidative stress, and the bone remodeling regulatory network. Therefore, interventions targeting abnormal subchondral bone remodeling, immunometabolic imbalance, and dysfunction of the osteochondral unit may provide new research directions for OP-OA comorbidity management, but more direct comorbidity studies are needed to validate their clinical significance (**Figure 5**).

**Figure 5:**
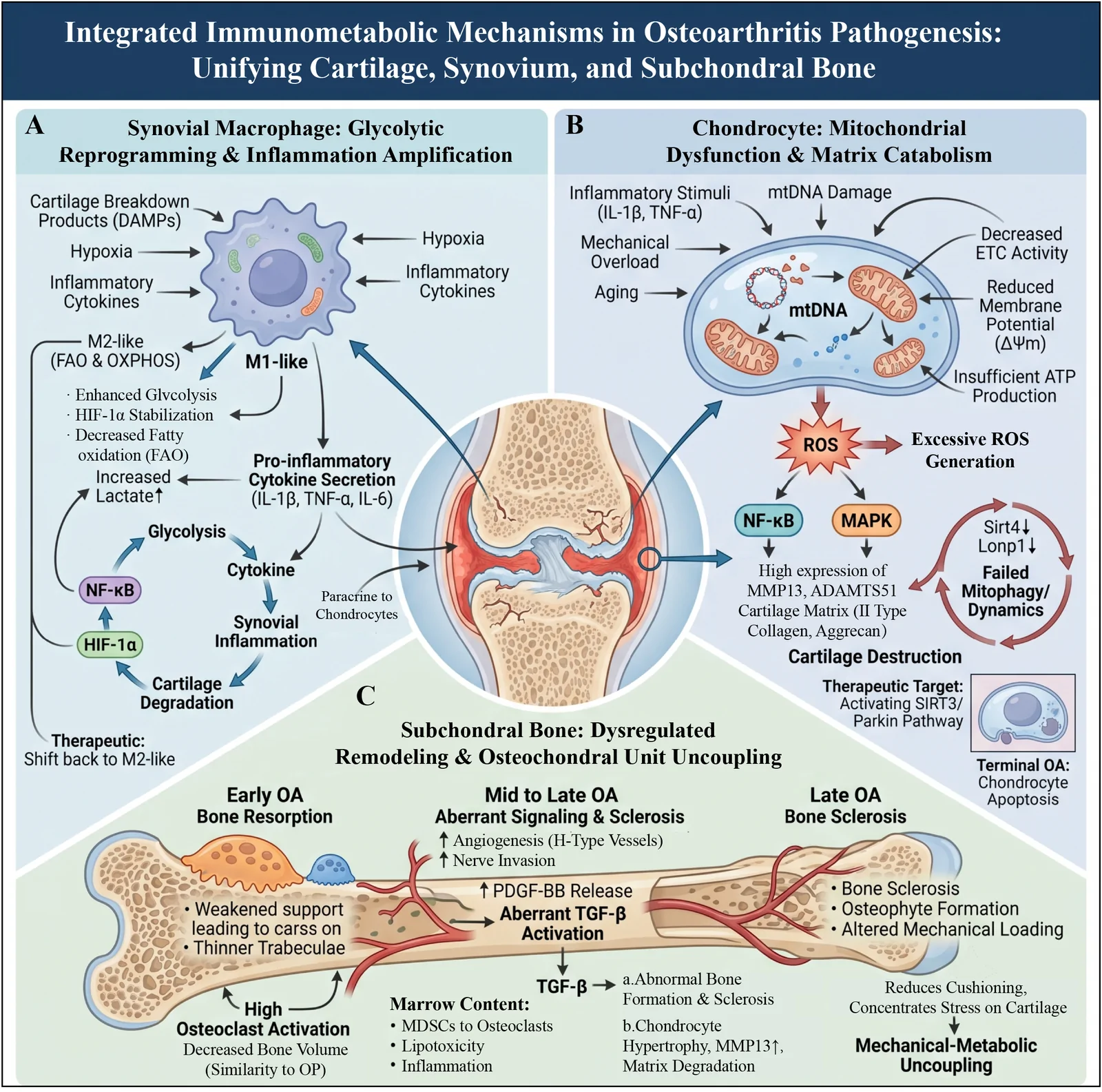
Immunometabolic integration mechanisms of cartilage, synovium, and subchondral bone in osteoarthritis pathogenesis. This figure outlines the immunometabolic crosstalk among the synovium, cartilage, and subchondral bone in OA. **(A)** Synovial macrophages undergo glycolytic reprogramming under stimulation by hypoxia, DAMPs, and inflammatory factors, transitioning to an M1-like pro-inflammatory phenotype and releasing IL-1β, TNF-α, and IL-6, amplifying synovial inflammation and cartilage degradation. **(B)** Chondrocyte mitochondrial dysfunction leads to increased ROS, insufficient ATP, failed mitophagy, and activation of NF-κB/MAPK, promoting MMP13 and ADAMTS5 expression and cartilage matrix destruction. **(C)** The subchondral bone undergoes early bone resorption and mid-to-late sclerosis, accompanied by abnormal TGF-β activation, H-type angiogenesis, neural invasion, and abnormal mechanical loading, ultimately driving osteochondral unit uncoupling, cartilage destruction, and OA progression. This image was drawn using Adobe Illustrator software.

## 7 Common mechanism network and therapeutic implications

### 7.1 Inflammaging-immune-metabolic dysregulation-bone-cartilage unit imbalance: a potential intervention framework

Inflammaging, as a significant upstream background of aging-related bone and joint degeneration, can drive the common progression of osteoporosis and osteoarthritis through chronic low-grade inflammation, SASP accumulation, immune cell functional decline, and metabolic homeostasis disruption (164). In this process, pro-inflammatory cytokines, ROS, DAMPs, and abnormal metabolites continuously act on the bone marrow, synovium, cartilage, and subchondral bone microenvironment, inducing functional and metabolic reprogramming in osteoclasts, osteoblasts, chondrocytes, and synovial macrophages (165). These dysregulations can promote enhanced bone resorption, insufficient bone formation, cartilage matrix degradation, and abnormal subchondral bone remodeling, thereby establishing a pathological feedback loop between bone mass loss and joint degeneration.

In bone tissue, inflammaging can promote osteoclast activation and inhibit osteoblast differentiation and mineralization through NF-κB, mTOR, RANKL/RANK, and oxidative stress-related signaling, leading to uncoupling of bone resorption and formation (166). In joint tissue, abnormal glucose and lipid metabolism, accumulation of advanced glycation end products, mitochondrial damage, and synovial inflammation can jointly induce enhanced catabolic activity in chondrocytes, promoting the expression of matrix-degrading enzymes such as MMPs and ADAMTS, thereby accelerating cartilage degeneration (167).

The bone-cartilage unit, within this closed loop, is both a damaged target and an amplifier of inflammation and metabolic disorders. Chondrocytes, osteocytes, and synovial cells, upon injury or during senescence, release DAMPs, pro-inflammatory mediators, and matrix degradation products, which can further activate macrophages, T cells, and osteoclasts, amplifying local inflammatory responses (168). Therefore, theoretically, targeting inflammaging, immune-metabolic abnormalities, and bone-cartilage unit homeostasis may provide novel avenues for research into the management of OP-OA comorbidity.

However, it must be emphasized that current evidence for these mechanisms primarily originates from single-disease studies on OP or OA, *in vitro* studies, and animal models, with direct validation in OP-OA comorbid populations remaining scarce. Thus, immune-metabolic reprogramming is currently more suitable as a potential mechanistic framework and intervention hypothesis rather than a clearly established clinical therapeutic target.

### 7.2 Potential directions targeting mitochondrial dysfunction, nad^+^ metabolism, and oxidative stress

Mitochondrial damage is a key node linking inflammaging, energy metabolism disorders, and bone-cartilage degeneration. When mitochondrial function is impaired, reduced efficiency of the electron transport chain, compromised ATP production, and the excessive accumulation of ROS can simultaneously inhibit osteoblast function, promote chondrocyte senescence and matrix degradation, and exacerbate inflammasome and NF-κB-related signaling (169, 170). Therefore, maintaining mitochondrial function and mitigating oxidative stress have become important research directions for immune-metabolic intervention.

Currently, mitochondrial-targeted antioxidant strategies such as MitoQ and SS-31 have demonstrated potential to reduce ROS levels, improve mitochondrial function, and alleviate cellular damage in various models of bone metabolic abnormalities and cartilage degeneration (171). Additionally, regulating mitochondrial quality control pathways such as PINK1/Parkin may improve cellular metabolic homeostasis by promoting the clearance of damaged mitochondria (172). However, these strategies are primarily derived from cell and animal experiments, and direct evidence regarding their efficacy, optimal therapeutic window, and long-term safety in human OP-OA comorbidity is still lacking.

Beyond mitochondrial protection, the NAD^+^/Sirtuins axis is also an important node in aging-related metabolic regulation. With aging, a decline in NAD^+^ levels can weaken the activity of deacetylases such as SIRT1 and SIRT3, affecting mitochondrial biogenesis, antioxidant defense, and inflammatory regulation (173, 174). Supplementation with NAD^+^ precursors, such as nicotinamide riboside (NR) and nicotinamide mononucleotide (NMN), may partially restore NAD^+^/Sirtuins axis activity, improve mitochondrial function, reduce oxidative stress, and inhibit inflammatory responses (175, 176). In OA models, NAD^+^ supplementation may inhibit p38 MAPK and NF-κB signaling by activating SIRT1, reducing the expression of inflammatory factors and matrix-degrading enzymes (177); in OP-related models, restoration of the NAD^+^/SIRT3 axis helps improve mitochondrial function and osteogenic differentiation ability of bone marrow mesenchymal stem cells (178). Furthermore, inhibiting NAD^+^ consumption-related pathways is also considered to have potential anti-aging and anti-inflammatory effects (179). Therefore, NAD^+^ metabolic regulation has certain mechanistic rationale and preclinical research value, but whether it can improve clinical outcomes in OP-OA comorbid patients still requires further validation.

### 7.3 Regulating glycolysis, lipid metabolism, and immune cell phenotypes

Glycolytic reprogramming is a key metabolic feature of immune cell activation and inflammatory phenotype formation. In OP and OA-related studies, glycolytic regulatory molecules such as HIF-1α, HK2, and LDHA have been implicated in macrophage activation, osteoclast differentiation, and chondrocyte metabolic changes (137). Interventions targeting these metabolic nodes have shown the potential to modulate inflammatory states and tissue damage in some experimental models. However, glycolysis is not only involved in pathological inflammatory responses but is also an important metabolic pathway for maintaining normal function in various cells. Therefore, whether pathways such as HIF-1α/HK2/LDHA can serve as therapeutic targets for OP-OA comorbidity still requires consideration of cell type, disease stage, and potential safety issues.

Abnormal lipid metabolism is also an important factor connecting systemic metabolic disorders and bone-cartilage degeneration. Obesity, hyperlipidemia, and aging-related lipid metabolic changes can lead to increased free fatty acids, accumulation of lipotoxic species, and bone marrow adiposity, affecting bone and joint tissue homeostasis (180). Mechanistically, pathways such as AMPK and PPAR can regulate fatty acid oxidation, mitochondrial function, and inflammatory responses, making them promising therapeutic targets worthy of further study (181). AMPK activation may help improve energy metabolism, promote mitochondrial homeostasis, and inhibit mTOR-related pro-inflammatory responses; PPAR-related regulation may affect osteoblast, chondrocyte, and immune cell function by improving lipid metabolism, reducing endoplasmic reticulum stress, and lipotoxic damage (182). Therefore, strategies targeting abnormal lipid metabolism should not be merely understood as “lipid-lowering” but rather as potential ways to improve the immune-metabolic microenvironment of the bone marrow, synovium, and subchondral bone.

Immune cell phenotype remodeling is a key step in translating metabolic interventions into tissue protective effects. M1-like macrophage polarization, Th17/Treg imbalance, and IL-17/RANKL axis activation can simultaneously promote synovial inflammation, osteoclastogenesis, and cartilage matrix degradation (183). Therefore, therapeutic strategies should shift from simply inhibiting inflammatory factors to regulating the metabolic state of immune cells. For example, reducing excessive glycolysis in macrophages while restoring fatty acid oxidation and mitochondrial function could promote the transition from an M1-like pro-inflammatory phenotype to an M2-like repair phenotype; whereas inhibiting glycolysis in Th17 cells or enhancing the metabolic stability of Tregs could reduce IL-17/RANKL-mediated bone resorption and cartilage degeneration (184). However, these mechanisms are primarily derived from single-disease studies on OP, OA, and other inflammatory disease models, and their role in human OP-OA comorbidity still requires further confirmation. Therefore, the goal of immune-metabolic intervention is not to completely suppress immune responses, but to restore the balance between inflammatory regulation, tissue repair, and metabolic homeostasis within the bone-cartilage unit while maintaining immune defense functions.

### 7.4 From mechanistic nodes to comprehensive intervention strategies: challenges in clinical translation

Based on the aforementioned mechanisms, immune-metabolic intervention offers new research directions for OP-OA comorbidity. Unlike conventional therapies targeting bone resorption, pain control, and functional improvement, immune-metabolic strategies focus on common pathological links such as inflammaging, mitochondrial dysfunction, metabolic abnormalities, and disrupted immune homeostasis. Therefore, in the future, they are more likely to serve as supplements to existing treatments rather than replacements. For example, in OP patients, modulating the inflammatory microenvironment and improving mitochondrial homeostasis may have complementary effects with anti-resorptive or bone-forming therapies (185); in OA patients, metabolic regulation strategies can be combined with exercise, weight management, anti-inflammatory therapy, and local interventions to improve bone-cartilage unit function (167). However, clinical studies on the combined application of immune-metabolic interventions with existing treatments are still very limited.

Notably, because immune-metabolic pathways are integral to maintaining the physiological functions of bone, cartilage, and immune cells, their therapeutic manipulation poses potential safety challenges. For example, glycolytic regulation needs to avoid affecting normal immune responses and tissue repair processes; mitochondrial function intervention must consider the differences in energy metabolism requirements among different cell types; and NAD^+^ metabolic regulation requires further clarification of the systemic effects that may result from long-term intervention (186). Therefore, broadly modulating metabolic pathways may not be the optimal strategy, and future research should focus more on cell-type specificity, tissue-targeted delivery, and disease-stage-dependent precise regulation.

Furthermore, gut microbiota modulation may serve as an important supplement to immune-metabolic interventions. Strategies such as exercise, dietary optimization, prebiotics, probiotics, and postbiotics may indirectly regulate the bone and joint immune microenvironment by improving intestinal barrier function, modulating microbial-derived metabolites, and influencing the Treg/Th17 balance and macrophage function (187). However, the efficacy of microbiota-based interventions is confounded by factors including age, dietary patterns, comorbidities, and chronic drug exposure, leading to substantial inter-individual variability in gut microbiota composition and immune-metabolic status. Therefore, future efforts should combine patient immune-metabolic characteristics, microbiota typing, and clinical phenotypes to achieve more precise microbiota regulation, rather than adopting a “one-size-fits-all” intervention model.

To date, evidence supporting immune-metabolic and microbiota interventions has been derived primarily from *in vitro* studies, animal models, and single-disease investigations of OP or OA. Although these studies provide important insights into the potential mechanisms of OP-OA comorbidity, they cannot yet directly prove that related interventions can improve clinical outcomes in real comorbid patients. Future research needs to combine longitudinal cohorts, multi-omics analysis, precise patient stratification, and clinical trials to further clarify the mode of action of key immune-metabolic nodes in human populations and evaluate the efficacy, safety, and applicability of different intervention strategies. Therefore, immune-metabolic reprogramming-related strategies should currently be regarded as promising research directions rather than established clinical treatment plans. With the development of comorbidity models, translational medicine research, and precision medicine, their application value in the comprehensive management of OP-OA comorbidity is expected to be further clarified in the future (**Table 2**).

**Table 2:** Evidence classification of potential immunometabolic interventions for OP–OA comorbidity.

Common mechanism node	Key pathological process	Potential intervention strategy	Expected effect on bone and joint	Evidence grade	Evidence stage and main limitations	Reference
Inflammaging and SASP accumulation	Senescent cells release SASP, ROS, and DAMPs, activating NF-κB, mTOR, and RANKL/RANK signaling, leading to chronic low-grade inflammation.	Senolytics, SASP inhibitors, and anti-inflammaging interventions.	Reduce osteoclast activation, alleviate osteoblast inhibition, mitigate synovial inflammation, and decrease cartilage matrix degradation.	Animal + cell; Limited human aging research; OP-OA indirect evidence.	Mainly cellular and animal studies; need to clarify target cells, therapeutic window, and long-term safety.	(169–172)
Mitochondrial damage and oxidative stress	OXPHOS impairment, ATP deficiency, ROS and mtDNA release, activating inflammasomes and NF-κB signaling.	MitoQ, SS-31, PINK1/Parkin or SIRT3/Parkin pathway activation.	Improve osteoblast energy metabolism, reduce chondrocyte senescence and inflammatory amplification.	Cell + Animal; Lack of evidence of human OP-OA.	Mostly preclinical evidence; tissue targeting, dosage, and long-term effects are not yet clear.	(173–176)
NAD+/ Sirtuins axis decline	A decline in NAD+ levels leads to reduced SIRT1/SIRT3 activity, impaired mitochondrial biogenesis, and exacerbated inflammation.	NR, NMN, CD38 or PARP1 inhibitors.	Enhance mitochondrial homeostasis and antioxidant capacity, promote osteogenesis, and reduce cartilage catabolism.	Animal + cell; Early research on human metabolism OP-OA; Indirect evidence.	Lack of clinical trials in OP–OA comorbid populations; bioavailability and long-term safety need evaluation.	(177–179)
Enhanced glycolysis and abnormal lactate signaling	HIF-1α, HK2, and LDHA activation, promoting pro-inflammatory metabolism in macrophages, Th17 cells, and osteoclasts.	HIF-1α, HK2, or LDHA modulation; lactate metabolism and local acidic environment intervention.	Inhibit synovial inflammation, IL-17/RANKL axis, and osteoclastogenesis, reduce abnormal subchondral bone remodeling.	Cell + Animal; It mainly comes from single-disease studies of OP/OA.	Broad inhibition of glycolysis may affect normal cells; therefore, cell- and stage-specific delivery is required.	(137)
Lipid metabolism disorder and lipotoxicity	Elevated FFA, bone marrow adiposity, endoplasmic reticulum stress, and mitochondrial damage, promoting local inflammation.	AMPK activation, PPAR regulation, fatty acid oxidation restoration, and lipotoxicity intervention.	Improve bone marrow and synovial metabolic environment, promote osteogenesis, and reduce chondrocyte damage.	Research related to human metabolism + animals + cells; OP-OA indirect evidence.	These interventions should not be equated with simple lipid-lowering strategies, as different PPAR subtypes may exert distinct effects on bone.	(180–182)
Immune cell phenotype imbalance	Increased M1-like macrophages, Th17/Treg imbalance, and activation of IL-17/RANKL axis.	Promote M2-like polarization, inhibit Th17 metabolism, enhance Treg stability.	Restore balance between inflammation resolution and tissue repair, reduce bone resorption and cartilage degradation.	Animal + cell; Limited human immunity research; Evidence of indirect comorbidity.	Immune phenotype is time- and tissue-dependent; excessive immunosuppression may affect repair.	(183, 184)
Multimodal lifestyle and metabolic intervention	Mechanical underloading, nutritional imbalance, and gut microbiota dysbiosis collectively maintain systemic inflammation.	Exercise, nutritional optimization, gut microbiota modulation, and repurposing of drugs such as metformin and statins.	Activate AMPK/PGC-1α, improve mitochondrial function, immune homeostasis, and bone-cartilage metabolism.	Human observation studies + animal studies + some clinical studies.	Most studies target single OP or OA; efficacy and applicable phenotypes in comorbid populations are unclear.	(187)

## 8 Challenges and future directions

### 8.1 Insufficient definition, clinical classification, and model construction for comorbidity

OP and OA often coexist in the elderly population, but unified definitions and clinical classification criteria for this comorbidity are currently lacking. OP can be defined based on BMD, fracture risk, bone turnover status, or bone microstructural changes, while OA can be assessed by imaging grading, clinical symptoms, pain severity, functional limitations, or joint replacement outcomes. The substantial variability in diagnostic criteria and inclusion conditions across different studies leads to significant heterogeneity in identifying comorbid populations, and also limits the comparability and clinical translation of research findings. Therefore, future efforts should clarify the basic definition of OP-OA comorbidity and delineate distinct clinical phenotypes such as “imaging-defined comorbidity,” “symptomatic comorbidity,” “high fracture risk with concurrent OA,” and “abnormal subchondral bone remodeling-type comorbidity.”

Existing clinical classifications largely evaluate OP and OA separately, with few attempts to identify comorbidity subtypes from the perspective of overall osteochondral unit imbalance. Relying solely on BMD or Kellgren-Lawrence grading fails to reflect the complex relationships among subchondral bone microstructure, bone turnover status, synovial inflammation, pain sensitization, and immunometabolic abnormalities. Future efforts should integrate BMD, fracture risk, bone turnover markers, OA imaging grading, subchondral bone structural parameters, pain and functional scores, synovial inflammation indicators, and immunometabolic markers to establish a stratification system applicable to elderly comorbid populations. Subsequently, these efforts should further explore comorbidity subtypes such as “high inflammation-low bone mass type,” “active subchondral bone remodeling type,” and “metabolic disorder-driven type.”

The development of animal models also represents a major bottleneck in current research. Existing OP and OA studies predominantly employ single-factor models, such as ovariectomized (OVX) models to simulate estrogen deficiency-induced bone loss, and destabilization of the medial meniscus (DMM), anterior cruciate ligament transection (ACLT), or monosodium iodoacetate (MIA) models to induce joint degeneration. These models typically use young adult animals with isolated pathological stimuli, making it difficult to simulate the complex state of concurrent inflammaging, immunosenescence, metabolic disorders, low bone mass, and joint degeneration seen in elderly individuals. Future research may adopt combined models such as “OVX+DMM/ACLT” or “OVX+DMM/ACLT+aging/high-fat diet/glucose metabolic abnormalities,” and integrate Micro-CT, histology, immunohistochemistry, flow cytometry, and metabolic parameters to evaluate overall osteochondral unit changes, thereby more reliably validating the causal role of immunometabolic reprogramming in OP-OA comorbidity.

### 8.2 Insufficient standardization and spatiotemporal dynamic evaluation of immunometabolic indicators

Immunometabolic reprogramming is an important mechanism explaining OP-OA comorbidity, yet current indicators still lack unified standards in terms of detection methods, sample sources, and data interpretation. Glycolysis-related indicators include glucose uptake, lactate, extracellular acidification rate (ECAR), and HK2 and lactate dehydrogenase A (LDHA) activities; mitochondrial function can be evaluated through oxygen consumption rate (OCR), adenosine triphosphate (ATP), mitochondrial membrane potential, mitochondrial reactive oxygen species (mtROS), mitochondrial DNA release, and mitophagy, also involving NAD+/NADH, lipidomics, metabolomics, inflammatory cytokines, and bone turnover markers. Due to substantial differences in sample types, detection platforms, and experimental conditions across studies, cross-study comparison and repeated validation of existing results remain limited.

Improving comparability first requires establishing a standardized detection framework applicable to osteochondral comorbidity. Serum primarily reflects systemic inflammatory and metabolic status, whereas synovial fluid is closer to the local synovial and cartilage microenvironment, and subchondral bone and bone marrow samples are more suitable for evaluating bone remodeling and osteoimmunometabolism. Therefore, sample sources should be clarified according to research objectives, with unified protocols for collection, storage, processing, and detection to reduce bias introduced by tissue ischemia, cell isolation, freeze-thaw cycles, and detection time windows. On this basis, a core indicator panel consisting of ECAR/OCR, lactate, NAD+/NADH, ATP, mtROS, mitochondrial membrane potential, key lipid metabolites, inflammatory cytokines, and bone turnover markers can be constructed, and its stability and clinical applicability validated through multicenter studies.

Beyond technical differences in detection, host exposure factors also represent an important and easily overlooked source of confounding in OP-OA immunometabolic research. This is particularly evident in gut microbiome studies, where age-related microbial alterations are often intertwined with dietary patterns, multimorbidity, and long-term medication use. Dietary fiber intake, antibiotics, proton pump inhibitors, hypoglycemic agents, anti-osteoporosis drugs, and other chronic medications may all alter gut microbial composition and metabolic functions, further influencing inflammatory status, immune cell phenotypes, and bone metabolism. Therefore, future studies should systematically record and, where possible, control for diet, medications, comorbid diseases, body weight, and other lifestyle factors, avoiding misinterpretation of host factor-induced microbiota changes as disease-specific features of OP-OA itself. Standardized collection and statistical adjustment of such variables will help improve the credibility of associations among microbiome, immunometabolic indicators, and bone mass and joint phenotypes.

OP-OA research also needs to fully account for the temporal and spatial heterogeneity of the disease. OA subchondral bone can exhibit markedly different remodeling characteristics across stages: the early stage is characterized by osteoclast activation, increased bone resorption, and trabecular bone loss; the middle stage is characterized by vascular/neural invasion, increased H-type vessels, and abnormal TGF-β signaling; and the late stage is characterized by subchondral bone sclerosis, osteophyte formation, and severe cartilage damage. Therefore, a single time point or single tissue sample is insufficient to fully reflect the immunometabolic status of OP-OA comorbidity. Future studies may combine longitudinal animal studies and prospective clinical cohorts, integrating single-cell sequencing, spatial transcriptomics, metabolomics, radiomics, and high-resolution Micro-CT/MRI to dissect immunometabolic changes across different disease stages and tissue regions, further identifying disease progression-related biomarkers and potential intervention windows.

### 8.3 From mechanistic research to stratified intervention and clinical translation

Although strategies targeting mitochondria, NAD+ metabolism, glycolysis, lipid metabolism, and immune cell phenotypes have demonstrated promise in cellular and animal experiments, their safety, efficacy, and target populations in patients with real-world OP-OA comorbidity remain insufficiently validated. Most current studies remain at the mechanistic exploration stage, lacking prospective cohorts and randomized controlled trials based on comorbidity classification. In particular, strategies such as MitoQ, SS-31, NR, NMN, glycolysis modulators, Sirtuin modulators, and AMPK/PPAR-related interventions still require clarification of their tissue targeting, dose windows, long-term safety, and dual effects on bone and cartilage.

Future clinical translation should shift from “single-disease treatment” to “comorbidity-stratified intervention.” First, risk stratification models incorporating clinical phenotypes, imaging, bone metabolism, inflammatory cytokines, and immunometabolic indicators must be developed to identify populations most likely to benefit from specific metabolic interventions. For example, for “high inflammation-low bone mass type” patients, anti-inflammatory, anti-resorptive, and SASP-regulating strategies may be prioritized; for “mitochondrial damage-dominant type” patients, mitochondrial-targeted antioxidants and NAD+ metabolic interventions may be explored; and for “metabolic disorder-driven type” patients, emphasis should be placed on weight management, exercise, nutrition, gut microbiota, and lipid metabolism regulation. Second, clinical endpoints should not be limited to BMD, pain scores, or radiographic progression, but should also incorporate fracture risk, cartilage structural changes, subchondral bone remodeling, synovial inflammation, functional improvement, and quality of life.

The synergistic potential of existing pharmacological and non-pharmacological interventions also warrants re-evaluation from an immunometabolic perspective. Exercise, nutritional interventions, and gut microbiota modulation may exert osteochondral protective effects by improving AMPK/PGC-1α signaling, mitochondrial function, short-chain fatty acid metabolism, and ameliorating chronic low-grade inflammation. Metformin, statins, anti-resorptive agents, cartilage-protective strategies, and certain anti-inflammatory or natural product interventions may also produce synergistic effects through modulation of glycolysis, lipid metabolism, mitochondrial homeostasis, and immune cell phenotypes. Overall, future research should establish a translational pathway centered on “precise comorbidity classification—standardized immunometabolic indicators—dynamic osteochondral unit assessment—stratified clinical intervention,” advancing OP-OA comorbidity prevention and treatment from experience-based management toward mechanism-guided precision intervention (**Table 3**).

**Table 3:** Main challenges, priority solutions, and translational directions for OP–OA comorbidity research.

Research area	Current main challenges	Priority solutions	Expected translational value
Comorbidity definition and clinical classification	Inconsistent diagnostic criteria for OP and OA; BMD, imaging, symptoms, and fracture risk are often evaluated separately.	Establish standardized definitions for imaging comorbidity, symptomatic comorbidity, high fracture risk combined with OA, and abnormal subchondral bone remodeling type.	Improve consistency of study subjects and comparability across different studies.
Comorbidity phenotype identification	Relying solely on BMD or Kellgren–Lawrence grading makes it difficult to reflect overall abnormalities of the osteochondral unit.	Integrate BMD, bone turnover, OA imaging, subchondral bone structure, pain function, synovial inflammation, and immune-metabolic markers.	Identify subtypes such as high inflammation–low bone mass type, active remodeling type, and metabolic disorder-driven type.
Animal model construction	Single models such as OVX, DMM, ACLT, or MIA fail to adequately recapitulate the multifactorial characteristics of elderly comorbidities.	Construct composite models combining OVX with DMM/ACLT, and superimpose aging, high-fat diet, or abnormal glucose metabolism.	More reliably verify the causal role of immune-metabolic reprogramming and osteochondral crosstalk.
Standardization of immune-metabolic indicators	Detection methods and sample sources for ECAR, OCR, lactate, NAD+/NADH, and mtROS vary widely.	Standardize sample collection, preservation, processing, and detection procedures; establish a core indicator set across cells, animals, and clinical samples.	Support multicenter comparisons, comorbidity classification, efficacy prediction, and target screening.
Sample and tissue levels	Serum is difficult to fully reflect the local microenvironment of joints, subchondral bone, and bone marrow.	Clarify the applicable scope of serum, synovial fluid, synovium, subchondral bone, and bone marrow samples, and conduct paired analysis.	Distinguish between systemic metabolic abnormalities and local osteochondral immune-metabolic changes.
Spatiotemporal dynamic evaluation	Most studies rely on single-time-point assessments, neglecting disease stage and osteochondral spatial heterogeneity.	Integrate data from longitudinal cohorts, single-cell sequencing, spatial transcriptomics, metabolomics, radiomics, and high-resolution imaging.	Analyze stage-specific changes and determine optimal intervention windows.
Stratified treatment and drug translation	Interventions targeting mitochondria, NAD+, glycolysis, and lipid metabolism are mostly at the preclinical stage.	Conduct stratified trials targeting mitochondria, Sirtuins, HIF-1α, AMPK/PPAR, and immune phenotypes based on comorbidity phenotypes.	Improve treatment matching and reduce off-target risks of broad metabolic inhibition.
Comprehensive intervention strategies	Existing studies mostly focus on single drugs or single diseases, lacking evaluation of dual benefits for bone and cartilage.	Combine exercise, nutrition, weight management, gut microbiota regulation, and drug repositioning strategies.	Simultaneously improve bone mass, mitigate cartilage degeneration, reduce inflammation, and restore metabolic status.
Clinical endpoint design	Using BMD, pain, or imaging progression alone is insufficient to reflect overall comorbidity benefits.	Simultaneously evaluate fractures, cartilage structure, subchondral bone remodeling, synovial inflammation, function, and quality of life.	Establish composite clinical endpoints oriented toward overall patient benefit.
Clinical validation and implementation	There is a scarcity of prospective cohorts, randomized controlled trials, and external validation.	Construct a multicenter translational pathway of “precise classification—indicator standardization—dynamic assessment—stratified intervention.”	Promote the transformation of OP–OA comorbidity from experience-based management to mechanism-guided precision intervention.

## 9 Conclusion

Immunometabolic reprogramming provides a potential integrative framework for understanding the multimorbidity of OP and OA. Existing evidence suggests that inflammaging, mitochondrial dysfunction, alterations in immune cell metabolism, and dysregulation within the osteochondral unit may jointly contribute to bone loss and joint degeneration. Metabolic changes such as enhanced glycolysis, impaired oxidative phosphorylation, abnormal lipid metabolism, and disruption of the NAD^+^/Sirtuins axis can influence bone remodeling, the inflammatory microenvironment, and tissue homeostasis by modulating the functions of osteoclasts, osteoblasts, chondrocytes, and synovial immune cells. Based on these insights, targeting immunometabolic abnormalities may pave the way for novel therapeutic strategies targeting OP–OA multimorbidity. However, current evidence is primarily derived from cell experiments, animal models, and single-disease studies of OP or OA, which largely highlight potential associations and provide mechanistic insights but do not yet establish a definitive causal role of specific metabolic pathways in human OP-OA multimorbidity. Future studies should integrate longitudinal cohorts, multimorbidity models, multi-omics approaches, and clinical intervention trials to further identify key regulatory nodes and their definitive pathological contributions. Future research should shift from single-disease and single-pathway analyses to a multimorbidity research paradigm centered on the overall homeostasis of the osteochondral unit. By establishing unified disease definitions, improving immunometabolic indicator systems, and employing precise stratification strategies, the metabolic characteristics and intervention windows of different patient subtypes can be elucidated, thereby advancing the management of OP-OA multimorbidity toward mechanism-guided precision prevention and treatment.

## Glossary

ACLT: anterior cruciate ligament transection
ADAMTS: a disintegrin and metalloproteinase with thrombospondin motifs
AMPK: AMP-activated protein kinase
ATP: adenosine triphosphate
BMD: bone mineral density
DAMPs: damage-associated molecular patterns
DMM: destabilization of the medial meniscus
ECAR: extracellular acidification rate
FFA: free fatty acids
FOXO: forkhead box O
GLUT1: glucose transporter 1
GPR81: G protein-coupled receptor 81
HIF-1α: hypoxia-inducible factor 1 alpha
HK2: hexokinase 2
JAK–STAT: Janus kinase–signal transducer and activator of transcription
LDHA: lactate dehydrogenase A
LONP1: Lon peptidase 1
LPS: lipopolysaccharide
MAPK: mitogen-activated protein kinase
MDSCs: myeloid-derived suppressor cells
MIA: monosodium iodoacetate
Micro-CT: micro-computed tomography
MMPs: matrix metalloproteinases
MitoQ: mitoquinone
MRI: magnetic resonance imaging
mtDNA: mitochondrial DNA
mtROS: mitochondrial reactive oxygen species
mTOR: mechanistic target of rapamycin
NADH: reduced nicotinamide adenine dinucleotide
NADPH: reduced nicotinamide adenine dinucleotide phosphate
NF-κB: nuclear factor kappa B
NFATc1: nuclear factor of activated T cells 1
NK: natural killer
NMN: nicotinamide mononucleotide
NR: nicotinamide riboside
OA: osteoarthritis
OCR: oxygen consumption rate
OP: osteoporosis
OPG: osteoprotegerin
OVX: ovariectomy
OXPHOS: oxidative phosphorylation
P1NP: procollagen type I N-terminal propeptide
PARP1: poly(ADP-ribose) polymerase 1
PDK4: pyruvate dehydrogenase kinase 4
PGC-1α: peroxisome proliferator-activated receptor gamma coactivator 1-alpha
PGE2: prostaglandin E2
PINK1: PTEN-induced kinase 1
PKM2: pyruvate kinase M2
PPAR: peroxisome proliferator-activated receptor
RANK: receptor activator of nuclear factor-κB
RANKL: receptor activator of nuclear factor-κB ligand
ROS: reactive oxygen species
RUNX2: runt-related transcription factor 2
SASP: senescence-associated secretory phenotype
SIRT1: sirtuin 1
SIRT3: sirtuin 3
SIRT4: sirtuin 4
SS-31: Szeto–Schiller peptide 31 (elamipretide)
TFAM: mitochondrial transcription factor A
TGF-β: transforming growth factor beta
Th17: T helper 17 cells
TLR4: Toll-like receptor 4
TNF-α: tumor necrosis factor alpha
Treg: regulatory T cells
